# Abundant Quantitative Trait Loci Exist for DNA Methylation and Gene Expression in Human Brain

**DOI:** 10.1371/journal.pgen.1000952

**Published:** 2010-05-13

**Authors:** J. Raphael Gibbs, Marcel P. van der Brug, Dena G. Hernandez, Bryan J. Traynor, Michael A. Nalls, Shiao-Lin Lai, Sampath Arepalli, Allissa Dillman, Ian P. Rafferty, Juan Troncoso, Robert Johnson, H. Ronald Zielke, Luigi Ferrucci, Dan L. Longo, Mark R. Cookson, Andrew B. Singleton

**Affiliations:** 1 Laboratory of Neurogenetics, National Institute on Aging, National Institutes of Health, Bethesda, Maryland, United States of America; 2 Department of Molecular Neuroscience and Reta Lila Weston Laboratories, Institute of Neurology, University College London, London, United Kingdom; 3 Department of Molecular and Integrative Neurosciences, The Scripps Research Institute, Jupiter, Florida, United States of America; 4 Division of Neuropathology, Department of Pathology, School of Medicine, Johns Hopkins University, Baltimore, Maryland, United States of America; 5 National Institute of Child Heath and Human Development Brain and Tissue Bank for Developmental Disorders, University of Maryland Medical School, Baltimore, Maryland, United States of America; 6 Clinical Research Branch, National Institute on Aging, Baltimore, Maryland, United States of America; 7 Lymphocyte Cell Biology Unit, Laboratory of Immunology, National Institute on Aging, National Institutes of Health, Baltimore, Maryland, United States of America; The Wellcome Trust Centre for Human Genetics, University of Oxford, United Kingdom

## Abstract

A fundamental challenge in the post-genome era is to understand and annotate the consequences of genetic variation, particularly within the context of human tissues. We present a set of integrated experiments that investigate the effects of common genetic variability on DNA methylation and mRNA expression in four human brain regions each from 150 individuals (600 samples total). We find an abundance of genetic *cis* regulation of mRNA expression and show for the first time abundant quantitative trait loci for DNA CpG methylation across the genome. We show peak enrichment for *cis* expression QTLs to be approximately 68,000 bp away from individual transcription start sites; however, the peak enrichment for *cis* CpG methylation QTLs is located much closer, only 45 bp from the CpG site in question. We observe that the largest magnitude quantitative trait loci occur across distinct brain tissues. Our analyses reveal that CpG methylation quantitative trait loci are more likely to occur for CpG sites outside of islands. Lastly, we show that while we can observe individual QTLs that appear to affect both the level of a transcript and a physically close CpG methylation site, these are quite rare. We believe these data, which we have made publicly available, will provide a critical step toward understanding the biological effects of genetic variation.

## Author Summary

In this paper, we describe a comprehensive assessment of the correlation between common genetic variability across the human genome, gene expression, and DNA methylation, within human brain. We studied the cerebellum, frontal cortex, temporal cortex, and pons regions of 150 individuals (600 tissue samples). In each tissue, we assessed 27,578 DNA methylation sites and the expression level of 22,184 genes. Our research shows that DNA methylation and RNA expression patterns differ between brain regions. Further, we show that DNA genotype is correlated with gene expression and DNA methylation, particularly when the genetic variation is close to the DNA methylation site or gene.

## Introduction

With the widespread application of highly parallel SNP genotyping arrays much of the recent effort in human genetics has focused on defining the role of genetic variation in disease and physical traits. A small subset of this work, however, has attempted to examine the more proximal effects of genetic variation on mRNA and protein levels [1]–[5]. This has the potential to inform on several levels; first, it is a critical step toward understanding the pathobiological consequences of genetic variants linked to disease; second, it affords the opportunity to form inferences regarding relationships between genes based on patterns of co-regulation; and third, it provides a more complete view of multiple levels of regulation of gene expression than that provided by the traditional reductionist method [6], [7].

Epigenetic alterations, including DNA methylation, histone modification and RNA mediated gene silencing, are defined as heritable changes in gene function that occur without an alteration of the underlying DNA sequence and which afford a level of transcriptional regulation above and beyond DNA sequence [8]. DNA methylation, which occurs at discrete CpG dinucleotide motifs, is believed to be an important mediator of gene expression; this observation has been most frequently linked to DNA methylation at CpG islands, regions of the genome that contain a high density of CpG sites, often proximal to gene promoter regions. A classical inverse relationship between the extent of DNA methylation at CpG islands and expression levels of the proximal gene product has been most often described [8]. To date the relationship between genetics, DNA methylation and gene expression is one that has been largely and necessarily confined to observations at single loci and transcripts in individual cell systems or tissues. The recent development of genome-scale technologies provides unprecedented opportunities to expand upon these experiments. The integration of genetic, epigenetic and expression data promises to provide general observations regarding the relationship between genetic variation and expression. Beyond these observations these data can be readily mined to unravel the network of effects associated with genomic variants. This may reveal some of the rather cryptic intermediate events that occur between DNA variant and phenotype.

Because of our interest in genomic regulation of expression and neurological disorders we embarked upon a series of experiments to provide a brain region-specific contextual framework for genetic and epigenetic regulation of gene expression. We were particularly interested in mapping the effects of common genetic variation on gene expression and DNA methylation; the widespread adoption of genome wide association studies for disease and traits has generated a large number of associated loci, and such a map would allow these loci to be associated with a biological consequence. We obtained frozen brain tissue from the cerebellum (CRBLM), frontal cortex (FCTX), pons (PONS) and temporal cortex (TCTX) from 150 subjects (total 600 tissue samples). We undertook three separate assays across this series; first, genome-wide SNP genotyping; second, assay of 27,578 CpG methylation sites in each of the four brain regions; third, mRNA expression profiling of 22,184 transcripts in all four brain regions. Here we discuss the results of these experiments, particularly in the context of integrated datasets to define expression quantitative trait loci (eQTL) and CpG methylation quantitative trait loci (methQTL), where quantitative trait loci (QTL) are correlations between genetic variation within genomic region(s) (in this case single nucleotide polymorphisms) and a quantitative trait (DNA methylation or expression), and detailing differences and similarities across brain regions.

## Results

### CpG methylation and mRNA levels differ between brain regions

**Figure 1:**
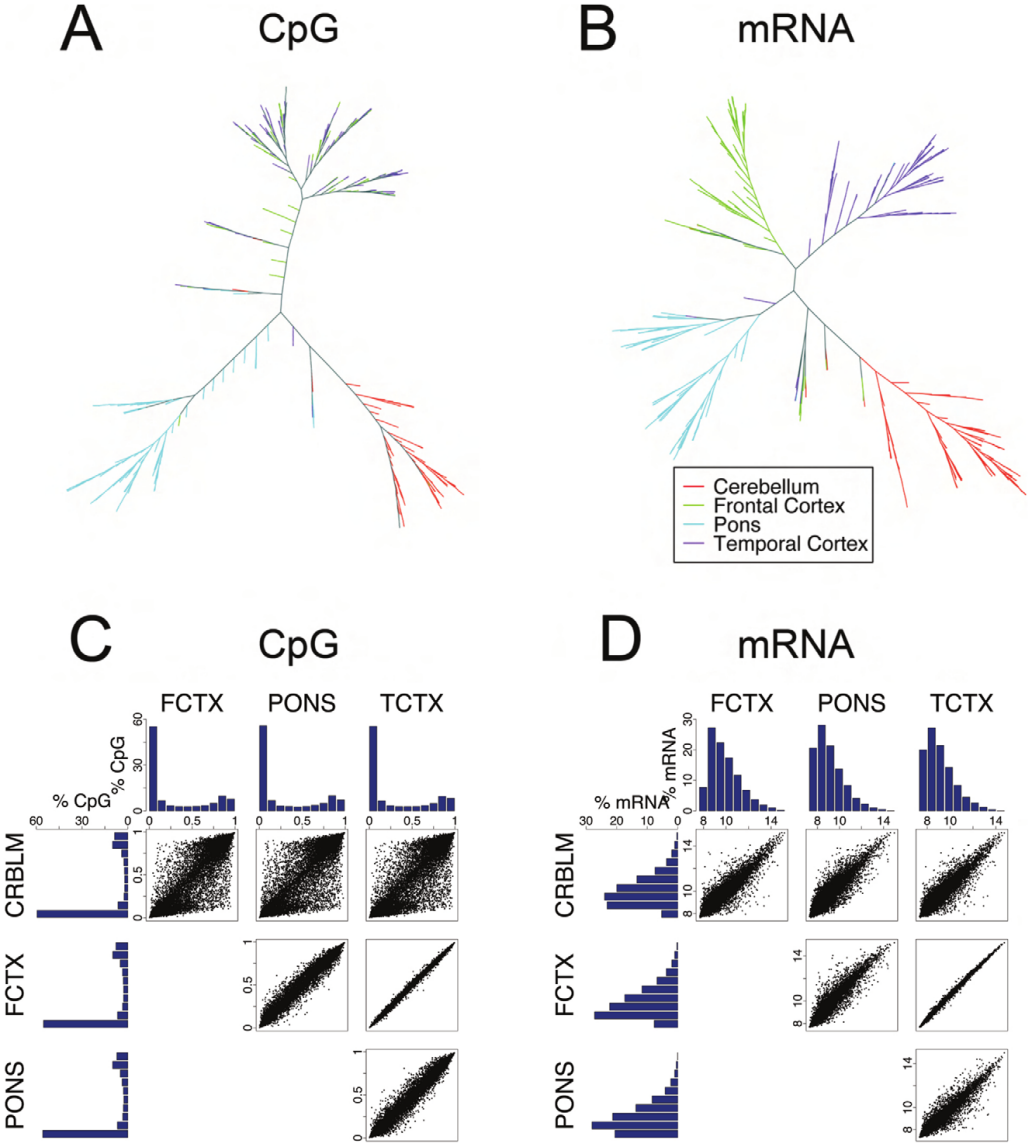
Analysis of CpG methylation and mRNA measures across four human brain regions. (A,B), unsupervised cluster analysis of CpG methylation levels at autosomal loci and mRNA expression levels. Data arising from each brain region are colored accordingly and demonstrate consistent separation of cerebellum, pons and cortical samples, with separation of frontal and temporal cortex samples using mRNA transcript levels. (C,D) Tissue based pairwise comparisons of CpG methylation and mRNA expression. The analyses in these figures used cleaned data. Histograms show the distribution of CpG methylation levels and mRNA expression levels for each tissue. Scatter plots are direct comparison of the level of each detected transcript or methylation level in each tissue pair. Notably frontal cortex and temporal cortex show the most similar patterns of CpG methylation and expression; conversely, all comparisons against cerebellar tissue show this tissue to have the most distinct patterns for all measures.

To assess whether differences in CpG methylation and expression were consistently different between brain regions, our initial analyses focused on global comparison of these measures across tissues. Performing a hierarchical cluster analysis [9] of these data demonstrated that the four brain regions have different epigenetic and expression profiles (Figure 1A and 1B). Expression pattern differences were distinct between cerebellum, pons and cortical tissue, with frontal and temporal cortices clearly separating within the mRNA dataset, but overlapping within the CpG methylation data. These data show that CpG methylation and mRNA levels vary measurably between brain regions. These data are in agreement with previous work that demonstrated distinct patterns of gene expression and DNA methylation in human cerebellum compared to cortical tissues [10], [11].

The next analysis was limited to the CpG methylation and mRNA datasets of those probes where an Illumina detection p-value of less than or equal to 0.01 was observed in 95% of samples analyzed in each of the four tissue region. This provided data on a total of 27,277 unique CpG methylation sites and 8,076 probes against individual mRNA transcripts that were present in all four brain regions (Figure S2). The distribution of observed CpG methylation levels and transcript abundance was plotted as a histogram for each tissue (Figure 1C and 1D). A high proportion of CpG sites interrogated within this assay were infrequently methylated, whereas a smaller proportion were highly methylated, a trend that was apparent across tissues. This distribution of DNA methylation (Figure 1C) closely matches distributions of DNA methylation reported previously by sequencing in multiple human cell types [12], [13]. CpG sites that are within CpG Islands [14], [15] were predominately unmethylated. Grouping of mRNAs into high through low expression groups revealed abundance profiles similar to those previously reported (Figure 1D). We next compared CpG methylation and mRNA expression levels at individual loci directly between each possible pair of tissue regions (Figure 1C and 1D, scatterplots). In general levels of DNA methylation and expression were quite similar between tissues. Measures within frontal and temporal cortices were consistently the most alike and cerebellar tissue provided the most distinct profile of the four regions.

### Genotype influences CpG methylation and mRNA expression

A primary aim underlying these experiments was to examine the extent of genetic control of DNA methylation and expression within brain tissues. To investigate this process, we undertook a series of QTL analyses. From the 537,411 genotyped SNPs that passed quality control filtering we then imputed genotypes for 2,545,178 SNPs using MACH [16] and HapMap CEU phase data. After additional quality and analysis specifications filtering of the imputed genotypes 1,629,853 SNPs (average) were used for analysis. The QTL analysis of each tissue region was performed separately so we expanded our trait selection from those that were detected in 95% of samples across all four brain regions to those that were detected in 95% of samples within a specific brain region. Changing this trait selection threshold based on what is present within a specific brain region allowed us to analyze additional mRNA transcripts that are well detected within one or more brain regions but not all four tissue regions. The number of SNPs, CpG sites and mRNA transcripts tested for each brain tissue can be found in Table 1. With these data for each tissue we then performed linear regression of allele dosage against each measure using CpG methylation or expression of mRNA as the dependent variable and genotype as the independent variable. This regression analysis was performed with Plink [17] to correlate allele dosage with the quantitative trait. We corrected for number of tests per trait by permutation, computing a genome-wide empirical p-value for each of the ∼1.6 million SNPs tested against each individual trait. To correct for the number of traits tested within each brain region by assay type an FDR threshold for significance was determined based on the empirical p-values from the proceeding step. This yielded a necessarily conservative threshold for significance [18]. Prior to analysis each trait was adjusted using available biological and methodological covariates in an attempt to reduce the influence of systematic confounding effects (Text S1). *Post hoc* we annotated significant QTLs as *cis* if the SNP lay within 1MB of either the CpG methylation site in question or the transcript being tested; all other SNP-dependent variable tests were designated as *trans*. Notably, because designation of *cis* and *trans* QTL tests was performed *post hoc*, there was no distinction in terms of level of statistical correction between these groups.

**Table 1:** Summary counts of number of samples, traits, and SNPs tested per tissue and assay type.

Counts of Tested Items								
	CpG				mRNA			
	CRBLM	FCTX	PONS	TCTX	CRBLM	FCTX	PONS	TCTX
Samples	108	133	125	127	143	143	142	144
Probes	27310	27532	27476	27538	8984	9842	8722	9372
SNPs	1540472	1624830	1602245	1607740	1653451	1653458	1650475	1655958

**Figure 2:**
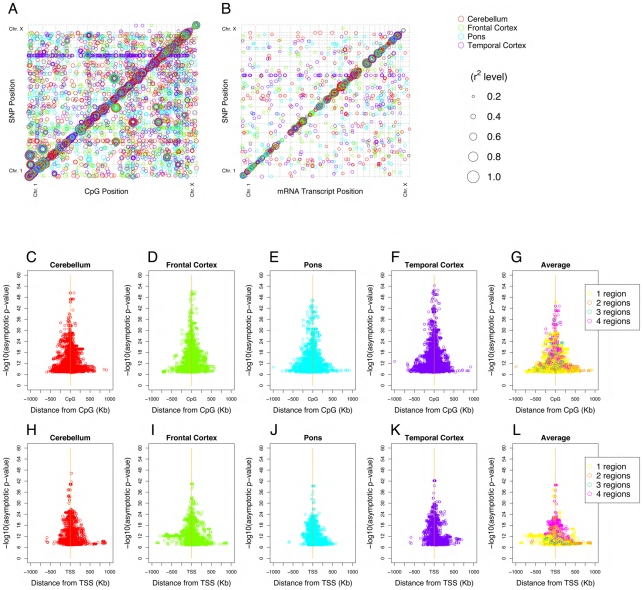
Quantitative trait loci (QTL) for CpG methylation (methQTL), mRNA expression (eQTL) in four brain regions. (A,B), methQTL and eQTL respectively, where the Y-axis represents the physical location of the QTL SNP and the X-axis the physical location of the QTL probe (CpG site or mRNA). The size of each point reflects the relative strength of the association (R^2^). (A) The excess of points along the diagonal illustrates that detected methQTL are predominantly *cis* acting; but that *trans* methQTLs with high R^2^ values exist across multiple tissues. (B) Shows the majority of eQTL loci are *in cis* and that the *in cis* effect sizes are larger that those observed *in trans*. (C–G) *cis* methQTL results showing a symmetric association between methylation level and variants on both sides of the CpG site in all four brain regions. (H–L) *cis* eQTL results showing a symmetric association between expression level and variants on both sides of the transcription start site (TSS, corrected for strand) in all four brain regions. G and L show the average p value for significant methQTL or eQTL across regions. For both methQTL and eQTL the more significant *cis* QTLs tended to be both closer to the transcriptions start site or CpG methylation site and common across tissue regions tested.

Our previous data from the human cerebral cortex [2], as well as data from HapMap lymphoblastoid cell lines (LCL) [5] have suggested that SNPs proximal to genes including SNPs upstream of the transcriptional start site (TSS) within the gene and downstream of the transcript end site (TES) have a greater influence on gene expression than those further away. This is presumably because genetic variation around promoter elements, splice sites and 3′ UTR affects transcription, splicing and mRNA stability [19] that results in a relative enrichment of *cis* over *trans* eQTLs. To see if this observation generalized to multiple brain regions and also to QTLs linked to CpG methylation, we plotted all significant QTLs by genomic position of the SNP and transcript or SNP and CpG site (Figure 2).

Previous work has shown that SNPs are weakly correlated with the methylation status of CpG islands as compared to other DNA characteristics such as sequence and structure [15]; however we detected a large number of significant correlations detected between genetic variation and the methylation status of CpG sites (Table 2). Genome-wide visualization of detected methQTLs illustrated a strong positional effect of this relationship with an excess of the number and magnitude of *cis* associations (Figure 2A). The detected methQTLs accounted for between 18% and 88% of corrected methylation levels at individual loci. The number of CpG sites with methQTLs that were significant after correction for genome-wide multiple testing ranged from 1085 (4%) in the cerebellum to 1417 (5.1%) in the temporal cortex (Table 2). All significant methQTL results can be found in Table S3. Detection of *cis* QTL for DNA methylation was more likely when the CpG site was outside of an island (as defined in [14]); while 42% of sites on the array are within a CpG island, only 18% of the CpG sites with a significant QTL were in islands.

**Table 2:** Summary counts of significant methQTL results found per brain tissue.

Significant QTL Counts for CpG sites												
	CRBLM			FCTX			PONS			TCTX		
methQTL	Pairs	CpGs	SNPs	Pairs	CpGs	SNPs	Pairs	CpGs	SNPs	Pairs	CpGs	SNPs
*cis*	9117	444	8458	9242	420	8402	7966	359	7103	12081	547	10875
*trans*	2985	657	2158	2893	740	2287	3408	774	2459	4653	886	2895
total	12102	1085	10606	12135	1153	10679	11374	1123	9536	16734	1417	13761

A similar number of mRNA transcripts with significant eQTLs were detected in each of the four brain regions, ranging from 280 (3.2%) in the pons to 391 (4.2%) in the temporal cortex (Table 3). These eQTLs accounted for between 18% and 77% of corrected expression levels of associated transcripts between individuals. All significant eQTL results can be found in Table S4.

**Table 3:** Summary counts of significant eQTL results (mRNA) found per brain tissue.

Significant QTL Counts for mRNA transcripts												
	CRBLM			FCTX			PONS			TCTX		
eQTL	Pairs	mRNAs	SNPs	Pairs	mRNAs	SNPs	Pairs	mRNAs	SNPs	Pairs	mRNAs	SNPs
*cis*	4053	147	4033	4781	167	4598	2944	102	2918	3509	141	3445
*trans*	1191	181	1079	734	170	612	471	179	369	1826	255	614
total	5244	319	4399	5515	334	5199	3415	280	3287	5335	391	4059

Proportionally a similar number of QTLs were observed for CpG methylation and mRNA expression. In order to determine whether SNP variability was more influential in CpG methylation or expression, we performed a random sampling, with 10,000 iterations, of QTL calculations for each independent measure in a core set of 100 individuals (Text S1). The results of this analysis show on average that 2.34% of CpG sites and 1.99% of mRNAs significantly correlate with QTLs. Each mRNA correlated with twice as many SNPs, on average, than CpG sites did. However the average R^2^ of the SNPs correlated with mRNAs was equal to CpGs (Figure S6).

The abundance of *cis* QTL for CpG methylation and mRNA expression prompted us to examine the distribution of *cis* methQTLs and eQTLs (Figure 2C–2L). This revealed that both the number of significant methQTLs and the strength of association between SNP and DNA methylation level were inversely correlated with physical distance between the genetic and epigenetic variants in question (Figure 2C–2F). Furthermore, this relationship was also evident for *cis* eQTLs (Figure 2H–2K). The average distance between correlated *cis* SNP and trait was 81Kb for CpG sites and 121Kb for mRNA transcripts. The largest effect QTLs for both *cis* methQTLs and eQTLs tended to be present in all four tissues tested (Figure 2G and 2L). Of the mRNA transcripts where a *cis* eQTL was significantly detected in at least one brain region 53% have been previously reported. This number increased to 70% when analysis is limited to those transcripts with a *cis* eQTL consistently detected in all four tissues [2]–[4] (Table S2).

**Figure 3:**
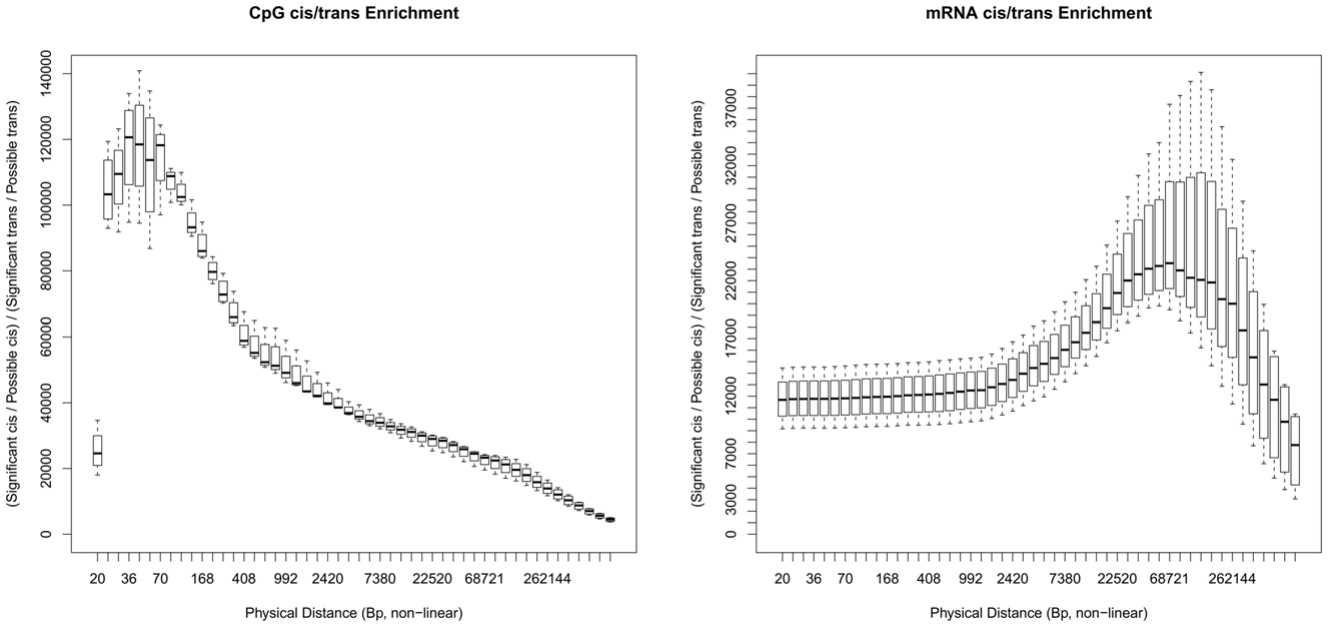
Peak enrichment for QTLs. Plots showing the large *cis* enrichment of significantly identified QTLs over *trans*. For the paper and result tables *cis* has been defined as SNPs and probes (CpG and mRNA) that are ±1 Mb of each other. At this annotated definition of *cis* the enrichment of results are on the right of these two plots x-axis, where the plots are all four brain tissue regions together. The left plot is for CpGs and the right for mRNA. At 1 Mb the enrichment of *cis* to *trans* for CpG QTLs is 4,427-fold and for mRNA is 7,255-fold. These plots show how this enrichment changes when considering other distances as a threshold for *cis*, the X-axis are these other distances (non-linear) at 50 distances that are four/fifths the size of the next larger distance. The plots are based on the proportions of significant *cis* and *trans* SNP/Probe pairings from the possible pairings at different distances.

To assess the enrichment of detected *cis* QTLs relative to those in *trans* we calculated the number of observed and possible *cis* and *trans* QTLs for CpG methylation and mRNA expression levels. The calculation of possible *cis* QTLs was performed by counting the number of all testable, genotyped SNPs within 1Mb of the CpG probe or transcript in question, regardless of significant association; calculation of possible *trans* QTLs was performed by counting the number of all testable, genotyped SNPs throughout the genome, excluding those within 1Mb of the CpG probe or transcript in question, again, regardless of significant association. This analysis was repeated for every tested CpG probe and transcript. These data showed an extreme enrichment of *cis* methylation and expression QTLs relative to trans (∼4400-fold and ∼7300-fold respectively). Although it should be noted that this calculation of enrichment does not take into account the differences in power to detect *cis* versus *trans* affects, thus the extreme nature of these fold changes may be inflated. While the average distance between correlated *cis* SNP and trait was 81Kb for CpG sites and 121Kb for mRNA transcripts when *cis* is defined as 1Mb, the peak enrichment of the number of significant *cis* QTLs was observed when the threshold distance for what is considered *cis* was set at ∼45bp for CpG sites and ∼68kb for mRNA transcripts (Figure 3).

### Large effect QTLs are consistent across brain regions

**Figure 4:**
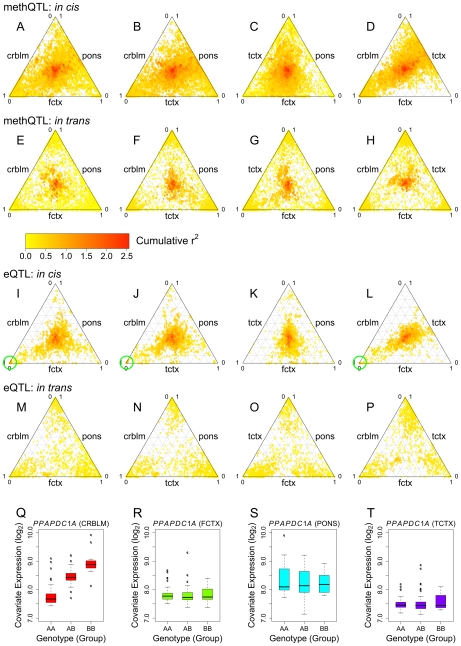
Comparison of QTLs for CpG methylation and mRNA expression across tissue regions. Any QTL that passed our threshold for significance in at least one tissue was included in the Ternary plots. The color of the points in the ternary plots reflects the cumulative R^2^ value for all tissues tested within each plot. Points toward the center indicate an equal R^2^ value across the three regions under investigation. Points toward the corner of a plot indicate a high R^2^ in one of the three tissues; points toward the edges of the plot indicate a high R^2^ in two of the three tissues. (A–H) Comparing methQTL in every three-way combination of the the four tissues for *cis* (A–D) and *trans* (E–H). (I–P) eQTL comparisons across each group of three tissues for *cis* and *trans* effects. Notably the cumulative R^2^ is generally higher for *cis* compared to *trans* loci across both methQTL and eQTL. Green circles highlight a cluster of relatively high cumulative R^2^ values driven primarily by the observed R^2^ within cerebellar tissue. These points were revealed to be a *cis* eQTL involving 20 SNPs and two neighboring transcripts, *PPAPDC1A* and *C10orf85*. (Q–T) Boxplots show expression level plotted against genotype for one of these eQTL SNP-transcript pairs (SNP rs2182513 and *PPAPDC1A*) and illustrate that this is a tissue specific QTL limited to the cerebellum.

Based on our results that reached statistical significance many QTLs appear to be tissue specific, where 49% of CpG sites and 54% of mRNA transcripts with a significant *cis* QTL were only detected within one tissue. Table 4 shows summary counts of CpG sites and mRNA transcripts with a QTL found in all four tissues. Because this analysis relies on a threshold for significance, it has the potential to be misleading, discounting QTLs that do not quite reach the threshold for significance. Thus, in order to compare detected methQTLs and eQTLs between tissues, we selected every SNP-CpG methylation pair and SNP-transcript pair that passed the defined threshold for significance in at least one tissue. We then compared R^2^ values for each of these SNP-CpG pairs or SNP-transcript pairs in all four tissues, including results from tissues where the SNP-CpG pair or SNP-transcript pair was non-significant, using ternary plots (Figure 4). The majority of large effect and many moderate effect QTLs were shared across the four brain regions (Figure 4) when significant effects from a tissue are compared with corresponding (possible non-significant) effects from another tissue. Of interest, a subset of *trans* methQTLs (defined here as any SNP CpG pair not within 1MB of each other) was also shared between regions and had high R^2^ values. This suggests that, while methQTLs show *cis* enrichment, there are also a number of robust distal effects where SNP and CpG methylation show significant association.

**Table 4:** Summary counts of significant QTL results per assay type identified that were present in all four tissues.

Significant QTL Counts Consistent across Tissues						
	CpG			mRNA		
QTLs	Pairs	Probes	SNPs	Pairs	Probes	SNPs
*cis*	2407	129	2336	1489	53	1489
*trans*	490	23	462	0	0	0
total	2897	152	2798	1489	53	1489

These plots illustrate that the majority of eQTLs or methQTLs with strong effect sizes were consistent across tissues. For example, a large effect eQTL was found for *CHURC1*, which encodes a protein proposed to be involved in transcriptional regulation, in all tissues (Figure S7 and as reported previously [3]). However, there were also rare, but observable, events where a large effect QTL was detected within a single tissue and was completely absent in the other three tissues. For example the *cis* eQTL for *PPAPDC1A*, encoding a phosphatidic acid phosphatase that displays hydrolase and phosphotase activity at the membrane, has a large effect that appears to be restricted to the cerebellum, despite reliable detection of the transcript in all four brain regions (Figure 4Q and 4R–4T and Figure S8).

### methQTL and eQTL capture independent traits

We next sought to investigate whether the observed methQTLs and eQTLs represent individual loci where the underlying variant influences both CpG methylation and gene expression. These data revealed that in general, there was little co-association between methQTLs and eQTLs. Of the SNP, CpG and mRNA combinations considered (see Materials and Methods), 4.8% (average across tissues) were significant as both a methQTL and eQTL; this includes 2.6% (average of 13 sites per tissue) of CpG methylation sites where a significant methQTL is present and 8.2% (average of 11 transcripts per tissue) of mRNA transcripts that have a significant eQTL.

**Figure 5:**
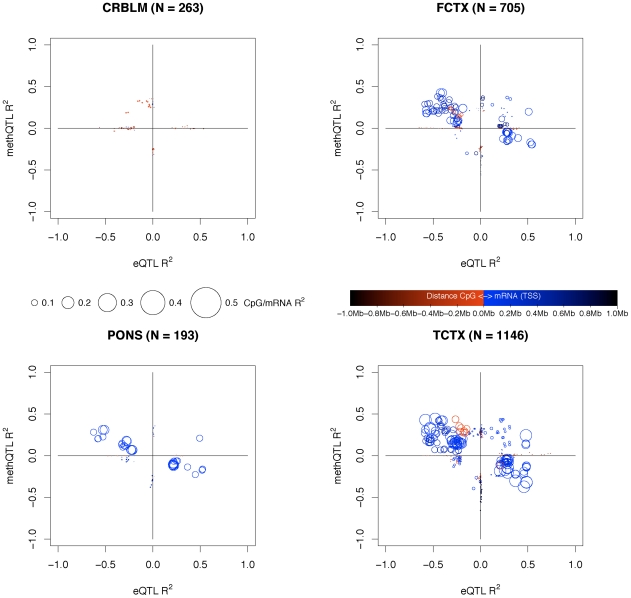
Intersection of QTLs for CpG and mRNA traits. Plots shown for each tissue region; cerebellum, frontal cortex, pons and temporal cortex. Per tissue for every pairing of CpG site and mRNA transcript where the CpG was within a CpG island and within 1Mb of the mRNA TSS and both the CpG and mRNA have a significant *cis* QTL; i.e. a triplet is formed between SNP, mRNA and CpG, where the SNP was significantly correlated in *cis* with either the CpG or mRNA of the CpG-mRNA *cis* pairs under consideration. For these triplets we plotted the eQTL R^2^ (X axis) and the methQTL R^2^ (Y axis). The R^2^ values are shifted into one of four quadrants (without changing the effect size) based on the positive and negative combinations of correlations for the eQTLs and methQTLs. A positive correlation with mRNA means that the level of expression is increased with the minor allele of the SNP and a negative correlation means that the mRNA expression level is decreased with the minor allele of the SNP. For CpGs a positive correlation implies that the level of methylation is increased at a CpG site with the minor allele and a negative correlation implies that the level of methylation is decreased with the minor allele. The top-left quadrant contains negative eQTL and positive methQTL correlations. The top-right quadrant contains eQTLs and methQTLs where the correlation was positive in both. The bottom-left quadrant contains eQTLs and methQTLs where the correlation was negative in both. The bottom-right quadrant contains positive eQTL and negative methQTL correlations. The triplets are plotted as circles where the radius of the circle represent the R^2^ value between the CpG site and mRNA transcript. The color of circle represents the distance between the CpG site and the mRNA TSS, where red indicates that the CpG site is close and upstream to the mRNA TSS; blue indicates that the CpG site is close to the mRNA TSS but downstream; and black indicates that the CpG site is farther from the mRNA TSS both up or downstream. Triplets that fall along the main diagonals are those where a significant methQTL and eQTL are present; the CpG and mRNA pairs for these triplets are closer together and have a larger R^2^ between the CpG and mRNA. Triplets that are on or close to the X axis are those where there is a significant eQTL but a lack of signal for the methQTL, likewise the items that are on or close to the Y axis are those with a significant methQTL and a lack of signal for the eQTL.

For this 4.8% with a shared QTL both the mean distance between the CpG site and mRNA TSS and the strength of the correlation differed from that of the considered combinations. For the shared QTLs the distance between CpG site and mRNA TSS is 27.5Kb whereas in all considered the mean distance was 394.4Kb. The mean R^2^ between CpG methylation and mRNA expression within the shared QTL set was 0.255 as compared to 0.033 in the considered set. Within these possibly shared QTLs, almost half of the significant methQTLs and eQTLs were correlated in the same direction, i.e. the CpG methylation level was positively correlated with the mRNA expression level (Figure S9). However; when considering only those shared QTLs where the CpG site was within a CpG island (as defined in [14]), 91% of these CpG methylation sites and mRNA expression pairs are inversely correlated, in line with the traditional view that increased CpG island methylation is associated with decreased expression at geographically local transcripts (Figure 5).

## Discussion

Elucidating the genetic control of biological processes is a critical issue in the post genome era. This will inform on the basic biological level and lead to clearer understanding of the pathophysiology underlying human disease. The work we have described here and the public release of the data resulting from this effort, aims to facilitate an understanding of the initial consequences of common genetic variation on DNA methylation and gene expression in brain (GEO Accession Number: GSE15745; dbGAP Study Accession: phs000249.v1.p1) [20]–[22].

Our data show clearly that patterns of expression are measurably different across brain tissues. QTL analyses reveal an abundance of eQTLs and, as previously reported, significant eQTLs are predominantly *cis* in nature [2], [5]. It is notable, particularly given the systematic differences in expression patterns among the four tissues, that the majority of large effect eQTLs detected were consistent across brain tissues. Large effect tissue-specific QTLs are also observable in the current data set, suggesting that there are some genetic effects on expression that are dependent on the tissue type used irrespective of expression levels of the mRNA. Our results that reached statistical significance are consistent with recent work showing that many eQTLs are likely tissue specific, but they typically have a smaller effect and are more distant [23]–[25]. However within our data when we observe a QTL that is only significant in a single tissue, examination of the correlation between the same SNP and trait in other tissues generally reveals a suggestive, although non-significant, correlation in the other tissues; thus both large and many moderate size effects appear to be shared. While this aspect of our data may seem at odds with the previous studies which in part used purified cells or cell lines, the current samples within our study were exclusively from heterogeneous tissues. Thus one might expect to see quantitative trait loci that are generalizeable across tissues because in essence each tissue is a heterogeneous mix of cells and we are more likely to detect QTLs that are in a majority of cell types. The detection of cell type specific QTLs is apparently still feasible, however, such correlations will have to be large enough to detect within the background of a heterogeneous tissue.

We have shown for the first time that there are large numbers of methQTLs in human tissue. It is worth noting, however, that the CpG methylation sites assayed here have not been defined through experimental work; as such a proportion of these may be consistently non-methylated, and thus our estimate of the proportion of CpG sites with an associated methQTL may be an underestimate. As for mRNA expression, DNA methylation patterns are sufficiently different to predict the originating tissue; however, we also see that SNP variation strongly influences the level of DNA methylation at sites that show intermediate levels of methylation. The strength of effect for methQTLs is similar to those for eQTLs and as discussed above eQTLs and methQTLs tend to be found consistently across tissues, supporting the robustness of these loci. Additionally methQTLs also show *cis*-enrichment, suggesting that local genomic variants influence the propensity of a particular genomic site to be methylated.

Combined analysis of methQTLs and eQTLs did not reveal a simple relationship between genetically influenced DNA methylation and expression. QTLs that influenced both DNA methylation and expression levels of a physically close transcript were identified, these were in the minority; for the most part strong methQTLs were not also QTLs for expression. It is plausible that this observation reflects a genuine biological disconnect between CpG methylation and gene expression. Alternatively, this may be a result of poor power within the series to detect such effects or, as discussed below, a consequence of differences in cellularity confounding our results. Notably, when analyzing joint methQTL-eQTLs where the methylation site in question resided in a CpG island, the classical inverse relationship between CpG methylation and expression was clearly observed.

Increasing the sample size of the current study will likely yield a larger number of QTLs, particularly those with small to moderate effect size. It would also be of interest to extend this initial work to other tissues, which will not only be important in defining tissue specific QTLs, but also will afford us the opportunity to examine QTLs at transcripts not typically detected in brain. Additionally, we have limited our sampling to a population that represents only a small component of worldwide human genetic diversity [26]. Adding tissues from other populations would provide an understanding of genetic control of expression in different groups and a framework for interpretation of GWAS results in these groups. Furthermore, inter-population comparison would help refine the basis by which differences in genetic architecture affect expression. We also note that our coverage of DNA modifications is currently incomplete as there are other epigenetic modifications, such as acetylation, that are not currently accessible to high throughput techniques, and many more methylation sites exist than assayed here. Whether genotypic differences influence other components of the epigenome is unknown. Finally, in a complex organ such as brain cellularity will confound analyses correlating measures that vary between cell types. Thus, CpG methylation to mRNA correlations pose a considerable challenge in heterogeneous tissues. In the context of human tissues this problem may be solved by selection of cell type by laser dissection or by culture and terminal differentiation of cohorts of pluripotent stem cells. Both solutions have considerable technical limitations at this time.

In summary, we show data that we argue convincingly demonstrates QTLs for DNA methylation and expression exist across human tissues. The data presented here provides an initial basis for understanding how genetic variance in humans influences epigenetic marks and expression; we argue that these observations may be useful in the understanding of gene contributions to human phenotypes. As well as providing a more complete model of the complex control of gene expression in the brain, our data may be useful in moving rapidly from locus to mechanism of disease.

## Materials and Methods

### Samples and assays

Frozen tissue samples of the cerebellum (CRBLM), frontal cortex (FCTX), caudal pons (PONS) and temporal cortex (TCTX) were obtained from 150 neurologically normal Caucasian subjects (Text S1). 100–200mg aliquots of frozen tissue were sub-dissected from each tissue from all 150 subjects resulting in 600 tissue samples and used for methylation assay and expression assays. Genotyping was performed using Infinium HumanHap550 beadchips (Illumina) to assay genotypes for 561466 SNPs, from the cerebellum tissue samples. CpG methylation status was determined using HumanMethylation27 BeadChips (Illumina), which measure methylation at 27,578 CpG dinucleotides at 14,495 genes. Profiling of 22,184 mRNA transcripts was performed using HumanRef-8 Expression BeadChips (Illumina) as previously described [27].

### Genotype data

The threshold call rate for inclusion of the subject in analysis was 95%. All 150 subjects had a call rate greater than 95%, and were included in the subsequent analyses (average call rate = 99.86%; range 97.72%–99.95%, based on the *missing* procedure within the PLINK v1.04 software toolset [17].

The gender of the subjects reported by the brain banks was compared against their genotypic gender using PLINK's *check-sex* algorithm, which determines a sample's genotypic gender based on heterozygosity across the X chromosome. A gender discrepancy was detected for one subject and excluded from further analysis.

To confirm the ethnicity of the samples, Identity-By-State (IBS) clustering and multidimensional scaling analyses were performed within PLINK using the genotypes from the brain samples that had been merged with data from the four HapMap [28] populations (n = 32 Caucasian (CEU), 12 Han Chinese, 16 Japanese and 24 Yoruban non-trio samples previously genotyped by Illumina and assayed on the Infinium HumanHap500 version genotyping chips). Two samples were outliers based on population and excluded from further analysis (Figure S1).

Genotype data of the samples were compared for cryptic relatedness using the Identity-By-Descent (IBD) procedure within PLINK. No samples were found to be from related individuals.

Mach software version 1.0.16 [16] and HapMap CEU phase data (release 22) were used to impute genotypes for ∼2.5 million SNPs. Imputed SNPs were excluded if the linkage disequilibrium r^2^ values between imputed and known genotypes was less than 0.3, and if their posterior probability averages were less than 0.8 for the most likely imputed genotype. For each of the four tissue regions, SNPs were also excluded if: (a) call rate was less than 95%, (b) Hardy-Weinberg equilibrium (HWE) p-value was less than 0.001, and (c) the SNP had less than 3 minor homozygotes present. Exact numbers of SNPs used per brain tissue and assay type are shown in Table 1.

### mRNA expression data

Raw intensity values for each probe were transformed using the rank invariant normalization method [29]–[31] and then log_2_ transformed for mRNA analysis. Four samples were excluded from further analysis, as they were outliers based on overall probe detection rate and/or mean expression level; one CRBLM, one FCTX and two PONS.

### CpG methylation data

The threshold call rate for inclusion of samples in the analysis was 95%. Based on this metric, 9 CRBLM, 2 FCTX, 6 PONS and 8 TCTX samples were excluded from further analysis. The remaining brain samples had an average detection rate of 99.84% (range 95.0% to 99.98%).

The gender of the samples reported by the brain banks were compared against their assayed gender based on values of methylation from CpG sites on the X chromosome. Four samples with gender discrepancies were detected and were removed from subsequent analysis; two CRBLM, one PONS and one TCTX. The resulting HCL sample tree based on all Chromosome X probes, after removal of these four individuals is shown in Figure S3.

### Clustering of samples by brain region

Performing a Hierarchical Clustering (HCL) [9] of the sample profiles using the TM4 MeV version 4.1.01 tool [32], with Euclidian distances and ‘Average Linkage clustering’ resulted in the samples separating fairly well by brain tissue region. Where in mRNA separation of samples into 4 clusters matching brain tissue region was clear. The frontal and temporal cortices could not be separated in within CpG data. For clustering all detected data was used for mRNA; only detected data for autosomal probes was used in clustering of the CpG data, otherwise sub-clusters based on gender appeared. The HCL samples trees were saved a Newick tree files and plotted again using the HyperTree tool (http://hypertree.sourceforge.net/).

### Selection of traits for analysis

Traits were excluded from analysis if they were detected in less than 95% of samples for each tissue region. For each tissue region and trait type (CpG and mRNA) the 95% threshold was determined using total number of analyzable samples for this pairing of region and trait. A probe is considered detected for a sample if the reported Illumina Detection p-value was less than or equal to 0.01. Numbers of analyzable samples per trait (or assay) type and region are available in Table 1 overlap of detected probes among brain tissues is shown as Venn diagram in Figure S2.

### Correction for known biological and methodological covariates

Prior to quantitative trait loci analysis each trait was adjusted using the available biological and methodological covariates in an attempt to remove the influence of these potentially confounding affects. In R each trait was regressed using the following model:
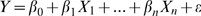
Where Y is the trait profile (log_2_ normalized mRNA expression intensities and raw values of CpG DNA methylation) and X_1_ … X_n_ represent the biological covariates Age and Gender and the methodological covariates post mortem interval (PMI), which Brain Bank the samples was from and which preparation/hybridization batch the sample was processed in. Within this model gender, tissue bank and batch where treated as categorical covariates. After fitting each trait to the model the residuals from the model are kept and represent the trait in following analyses. Thus variance attributable to gender, age, post-mortem interval, tissue source and hybridization batch are removed prior to QTL analysis. These covariate data are available in Table S1. Histograms showing the proportion of traits that are potentially impacted by these covariates are shown in Figure S4 (CpG) and Figure S5 (mRNA).

### Quantitative trait loci analysis

For each of the four brain regions, a regression analysis was performed on the residuals described in the preceding section for (a) mRNA expression and (b) the methylation values generated for CpG sites. The trait residuals were then used as the quantitative phenotype for that probe in genome-wide association analysis looking for quantitative trait loci. These analyses were performed using the *assoc* function within Plink, which correlates allele dosage with change in the trait. Each of the four tissue regions was analyzed separately, and independent genome-wide association analyses were performed looking for (a) expression quantitative trait loci (eQTLs) for mRNA and (b) methylation quantitative trait loci (methQTLs) for CpG sites. The Plink toolset quantitative trait association analysis fits data to the following model:
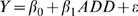
Where Y is the quantitative trait and ADD represent genotypes encoded as allele dosage. See Plink Quantitative trait association and Linear and Logistic models documentation for more information.

### Correction for multiple tests

To correct for the large number of SNPs tested per trait, a genome-wide empirical p-value was computed for the asymptotic p-value for each SNP by using 1,000 permutations of swapping sample labels of the traits, using the maxT permutation functionality provided within Plink. A permutation based method using label swapping of the traits is an appropriate method of test correction [18] for these analysis as it is not dependent on these quantitative traits having a normal distribution and also allows the linkage disequilibrium of the genomic regions being tested against the traits to be maintained.

To correct for the number of traits being tested per tissue region, a false discovery rate (FDR) threshold was determined based on the empirical p-values using the *fwer2fdr* function of the multtest package in R version 2.6.1. Empirical p-values were allowed to exceed this threshold if their linkage disequilibrium r^2^ was greater than or equal to 0.7 with a SNP with empirical values within the FDR threshold.

### Polymorphism(s) in assay probes

Sequence variants within the sequence of the probe used to assay individual traits may cause differential hybridization and inaccurate expression and possible methylation measurements. To exclude this confound, the sequences of probes with significant correlation to a trait were examined for the presence of polymorphisms using CEU HapMap data, and, if present, that QTL was removed from the result set.

### Selecting SNPs, CpG sites, and mRNA transcripts for shared QTL analysis

Within each tissue, we selected every pairing of CpG methylation sites and mRNA transcripts where the CpG methylation site was within 1Mb of the mRNA TSS and both the CpG methylation site or mRNA transcript had a significant *cis* QTL. These CpG-mRNA pairs were then expanded into triplets of SNP and CpG-mRNA pair, where the SNP was significantly correlated in *cis* with either the CpG methylation site or mRNA transcript of the CpG-mRNA pair.

### Accession numbers

Data resulting from these experiments is available online (GEO Accession Number: GSE15745; dbGAP Study Accession: phs000249.v1.p1).

## Supporting Information

Figure S1 Population MDS plot based on genotype from genome wide Identity-By-State pairwise distances between the 150 samples used in this study (LNG) and HapMap samples (CEU, CHB, JPT and YRI). The plot shows that of the 150 samples originating from reported Causasian individuals from the United States, two samples (indicated by Removed labels) are ethnic outliers relative to the LNG cohort.
(0.02 MB PDF)

Figure S2 Venn diagrams showing the frequency overlap of the number of probes that are detected in 95% of samples between the four brain tissues. Venn diagrams are shown for both CpG and mRNA assay types. The rectangles with different orientations and border, shown on the left legend represent the different tissue and the different squares represent overlapping frequencies between different tissues. The colored squares represent the number of tissues overlapping, where the central blue square in each Venn diagram represent the number of probes reliably detected in all four tissues. Hence, the blue square in the mRNA diagram indicates that 78.2% of the 10326 mRNA probes detected in at least one tissue region were also detected in all four tissues regions or 8076 probes.
(0.01 MB PDF)

Figure S3 This tree shows that male and female samples (using all brain tissue regions) separate based on gender if a hierarchical cluster is performed on CpG methylation data for only Chromosome X CpG sites. The plot was generated in HypterTree using the samples tree generated from an HCL of Chromosome X methylation data using ‘Average Linkage clustering’. The plot is after removal of the 4 individuals that appeared to be gender mismatches.
(0.04 MB PDF)

Figure S4 Histograms of potential covariate effects on CpG DNA methylation levels, if the data had not been adjusted for these confounds prior to QTL analysis. Each row of sub-plots represents a single covariate and each column represents a brain tissue region; CRBLM (red), FCTX (green), PONS (blue) and TCTX (purple). The histograms show the number of probes (CpG sites) on the y-axis and the R^2^ values from the regression of the covariates with each probe.
(0.04 MB PDF)

Figure S5 Histograms of potential covariate effects on mRNA expression levels, if the data had not been adjusted for these confounds prior to QTL analysis. Each row of sub-plots represents a single covariate and each column represents a brain tissue region; CRBLM (red), FCTX (green), PONS (blue) and TCTX (purple). The histograms show the number of probes (mRNA transcripts) on the y-axis and the R^2^ values from the regression of the covariates with each probe.
(0.04 MB PDF)

Figure S6 Histograms representing the results of the resampling analysis. Each row represents a different assay type (CpG and mRNA) and each column represents a different metric of the resampling; number of probes, average number of SNPs per probe and average R^2^ of probe/SNP correlations.
(0.11 MB PDF)

Figure S7 These plots, one for each brain tissue, show the p-values of correlations between SNPs and mRNA transcripts in a 500Kb region centered on CHURC1. Where a eQTL in this genomic region is present in all four brain tissues. A cis QTL for CHURC1 has also been reported within liver (Schadt et al., 2008). Within each plot the X-axis is the physical position along this region of the chromosome and the Y-axix is the −log_10_(asymptotic p-values) for the correlations. The p-values are colored and numbered to match the annotated transcripts labeled in the top portion of the plots. Thus in CRBLM, PONS and TCTX the dark blue ‘5’s are p-values for CHURC1 and in FCTX the dark red ‘6’s are p-values for CHURC1. mRNA transcript annotations shown in grey are those where a probe in present on the expression platform but are not detected in 95% of the tissue.
(0.43 MB PDF)

Figure S8 These plots show an example of a cis eQTL for an mRNA that appears to be tissue specific. The plots, one for each brain tissue, show the p-values of correlations between SNPs and mRNA transcripts in a 1Mb region centered on PPAPDC1. Within each plot the X-axis is the physical position along this region of the chromosome and the Y-axix is the −log_10_(asymptotic p-values) for the correlations. The p-values are colored and numbered to match the annotated transcripts labeled in the top portion of the plots. Thus in CRBLM the red ‘2’s are p-values for SNPs correlated with PPAPDC1A. Also present at this same genomic locus is another tissue specific eQTL for the mRNA transcript C10orf85, shown as green ‘3’s.
(0.51 MB PDF)

Figure S9 Intersection of QTLs for CpG and mRNA traits. Plots shown for each tissue region; cerebellum, frontal cortex, pons and temporal cortex. Per tissue for every pairing of CpG site and mRNA transcript where the CpG was within 1Mb of the mRNA TSS and both the CpG and mRNA have a significant cis QTL; i.e., a triplet is formed between SNP, mRNA and CpG, where the SNP was significantly correlated in cis with either the CpG or mRNA of the CpG-mRNA cis pairs under consideration. For these triplets we plotted the eQTL R2 (X axis) and the methQTL R2 (Y axis). The R2 values are shifted into one of four quadrants (without changing the effect size) based on the positive and negative combinations of correlations for the eQTLs and methQTLs. A positive correlation with mRNA means that the level of expression is increased with the minor allele of the SNP and a negative correlation means that the mRNA expression level is decreased with the minor allele of the SNP. For CpGs a positive correlation implies that the level of methylation is increased at a CpG site with the minor allele and a negative correlation implies that the level of methylation is decreased with the minor allele. The top-left quadrant contains negative eQTL and positive methQTL correlations. The top-right quadrant contains contains eQTLs and methQTLs where the correlation was positive in both. The bottom-left quadrant contains eQTLs and methQTLs where the correlation was negative in both. The bottom-right quadrant contains positive eQTL and negative methQTL correlations. The triplets are plotted as circles where the radius of the circle represent the R2 value between the CpG site and mRNA transcript. The color of circle represents the distance between the CpG site and the mRNA TSS, where red indicates that the CpG site is closer upstream to the mRNA TSS; blue indicates that the CpG site is close the the mRNA TSS but downstream; and black indicates that the CpG site is farther from the mRNA TSS both up or downstream. Triplets that fall along the main diagonals are those where a significant methQTL and eQTL are present; the CpG and mRNA pairs for these triplets are closer together and have a larger R2 between the CpG and mRNA. Triplets that are on or close to the X axis are those where there is a significant eQTL but a lack of signal for the methQTL, likewise the items that are on or close to the Y axis are those with a significant methQTL and a lack of signal for the eQTL.
(1.74 MB PDF)

Table S1 Sample information, table containing the sample Id, originating brain bank, age, gender, postmortem interval, preparation/hybridization batch and cause of death where available.
(0.04 MB XLS)

Table S2 Table showing which mRNA transcripts indentified as having an eQTL within the current study have also been identified in another genome wide eQTL study.
(0.13 MB XLS)

Table S3 Significant QTL results from the analysis of CpG sites and SNPs for each brain tissue (CRBLM, FCTX, PONS, TCTX). Table includes which tissue analysis the result is from (Tissue); the Illumina ID for the CpG site (IlmnID); the chromosome and position of the CpG site (Chr, Start_Pos and Stop_Pos); the gene that has a transcription start site downstream and proximal the GpG site (Symbol, GeneID and Strand); the SNP correlated with the CpG site and its position (SNP, SNP_Chr and SNP_Position); the gene closest to the SNP that is correlated with the CpG site (Dist_SNP_Closest_Gene, SNP_Gene and SNP_GeneID); the Hardy-Weinberg Equilibrium p-value (HWE) for the SNP based on the samples used in the analysis for the specified tissue (Tissue); the minor allele frequency (MAF) for the SNP based on the samples used in the analysis for the specified tissue (Tissue); the call rate for the SNP (SNP_Call_Rate) based on the samples used in the analysis for the specified tissue (Tissue); the fraction of samples used in the correlation between the SNP and CpG site based on the number of samples used in the analysis for the specified tissue (Tissue) where samples with missing genotypes or missing trait value are not included in the analysis; annotations for the SNP and CpG site are *cis* or *trans* where *cis* is +/− 1Mb (Dist_to_Trait and cis_trans); the asymptotic p-value, empirical p-value, regression r-squared and regression beta for the correlation (pvalue, empP, r2 and beta) note the empirical p-values equal to 0.000999 in the table represent p-values less than 0.001 (based on 1K permutations); and the number of AA, AB and BB genotypes used in the correlation (Num_AA, Num_AB and Num_BB).
(8.42 MB TXT)

Table S4 Significant QTL results from the analysis of mRNA transcripts and SNPs for each brain tissue (CRBLM, FCTX, PONS, TCTX). Table includes which tissue analysis the result is from (Tissue); the Illumina ID for the mRNA transcript (IlmnID); position and annotations for the mRNA transcript (Symbol, GeneID, Description, Chr, Start_Pos, Stop_Pos and Strand); the SNP correlated with the mRNA transcript and its position (SNP, SNP_Chr and SNP_Position); the gene closest to the SNP that is correlated with the mRNA transcript (Dist_SNP_Closest_Gene, SNP_Gene and SNP_GeneID); the Hardy-Weinberg Equilibrium p-value (HWE) for the SNP based on the samples used in the analysis for the specified tissue (Tissue); the minor allele frequency (MAF) for the SNP based on the samples used in the analysis for the specified tissue (Tissue); the call rate for the SNP (SNP_Call_Rate) based on the samples used in the analysis for the specified tissue (Tissue); the fraction of samples used in the correlation between the SNP and mRNA transcript based on the number of samples used in the analysis for the specified tissue (Tissue) where samples with missing genotypes or missing trait value are not included in the analysis; annotations for the SNP and mRNA transcript are *cis* or *trans* where *cis* is ±1 Mb (Dist_to_Trait and cis_trans); the asymptotic p-value, empirical p-value, regression r-squared and regression beta for the correlation (pvalue, empP, r2 and beta) note the empirical p-values equal to 0.000999 in the table represent p-values less than 0.001 (based on 1K permutations); and the number of AA, AB and BB genotypes used in the correlation (Num_AA, Num_AB and Num_BB).
(7.79 MB XLS)

Text S1 Genetic control of DNA methylation and expression across human brain tissues.
(0.06 MB DOC)
