# Supplementary figures and images for: Abundant Quantitative Trait Loci Exist for DNA Methylation and Gene Expression in Human Brain
Source: PLoS Genet. 2010 May 13;6(5):e1000952. doi: 10.1371/journal.pgen.1000952 (PMC2869317; doi:10.1371/journal.pgen.1000952)

Population MDS by Genotype

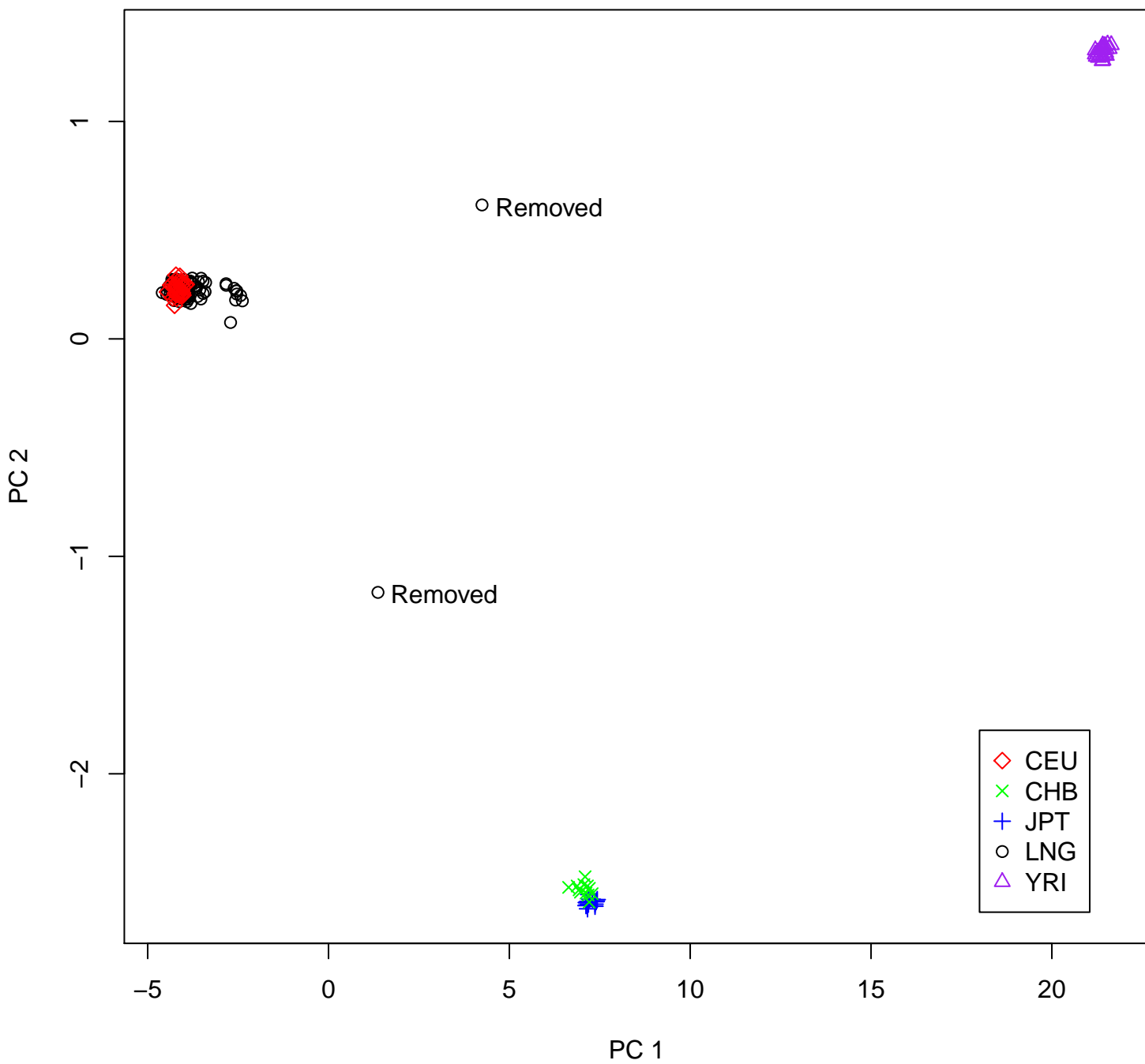

Supplement: Figure S1 — Population MDS plot based on genotype from genome wide Identity-By-State pairwise distances between the 150 samples used in this study (LNG) and HapMap samples (CEU, CHB, JPT and YRI). The plot shows that of the 150 samples originating from reported Causasian individuals from the United States, two samples (indicated by Removed labels) are ethnic outliers relative to the LNG cohort. (0.02 MB PDF) [file pgen.1000952.s001.pdf]

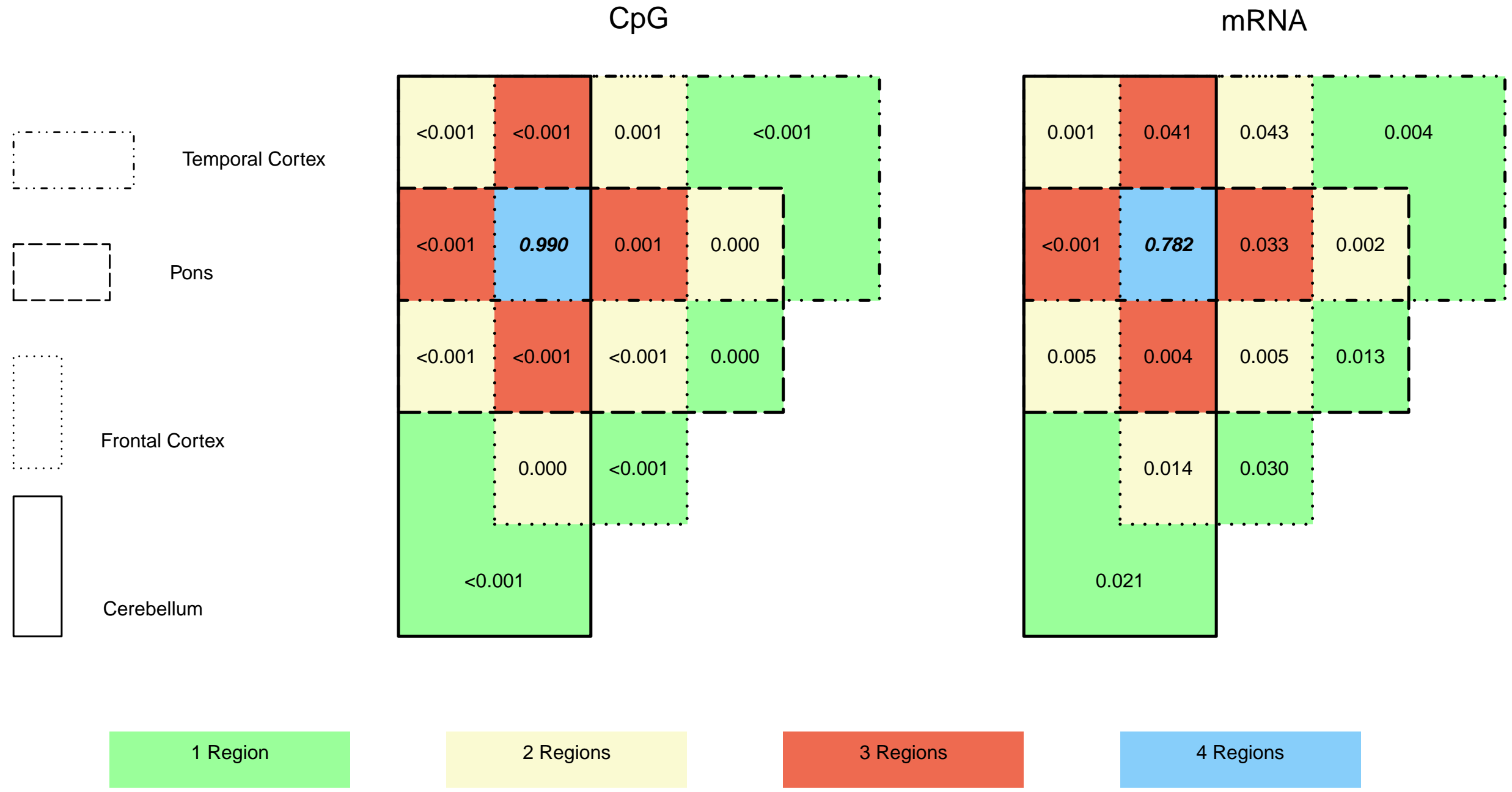

Supplement: Figure S2 — Venn diagrams showing the frequency overlap of the number of probes that are detected in 95% of samples between the four brain tissues. Venn diagrams are shown for both CpG and mRNA assay types. The rectangles with different orientations and border, shown on the left legend represent the different tissue and the different squares represent overlapping frequencies between different tissues. The colored squares represent the number of tissues overlapping, where the central blue square in each Venn diagram represent the number of probes reliably detected in all four tissues. Hence, the blue square in the mRNA diagram indicates that 78.2% of the 10326 mRNA probes detected in at least one tissue region were also detected in all four tissues regions or 8076 probes. (0.01 MB PDF) [file pgen.1000952.s002.pdf]

— Males  
— Females

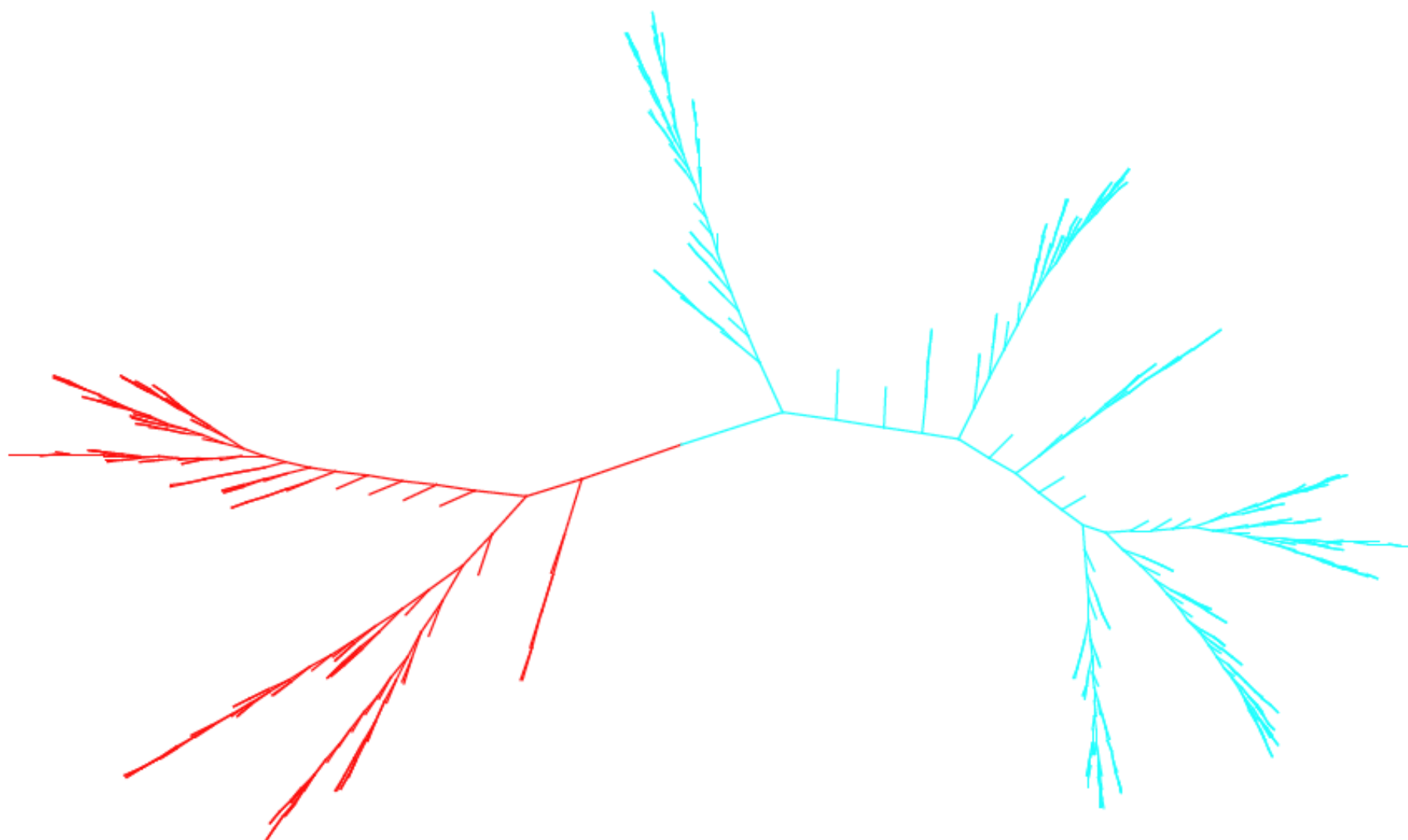

Supplement: Figure S3 — This tree shows that male and female samples (using all brain tissue regions) separate based on gender if a hierarchical cluster is performed on CpG methylation data for only Chromosome X CpG sites. The plot was generated in HypterTree using the samples tree generated from an HCL of Chromosome X methylation data using ‘Average Linkage clustering’. The plot is after removal of the 4 individuals that appeared to be gender mismatches. (0.04 MB PDF) [file pgen.1000952.s003.pdf]

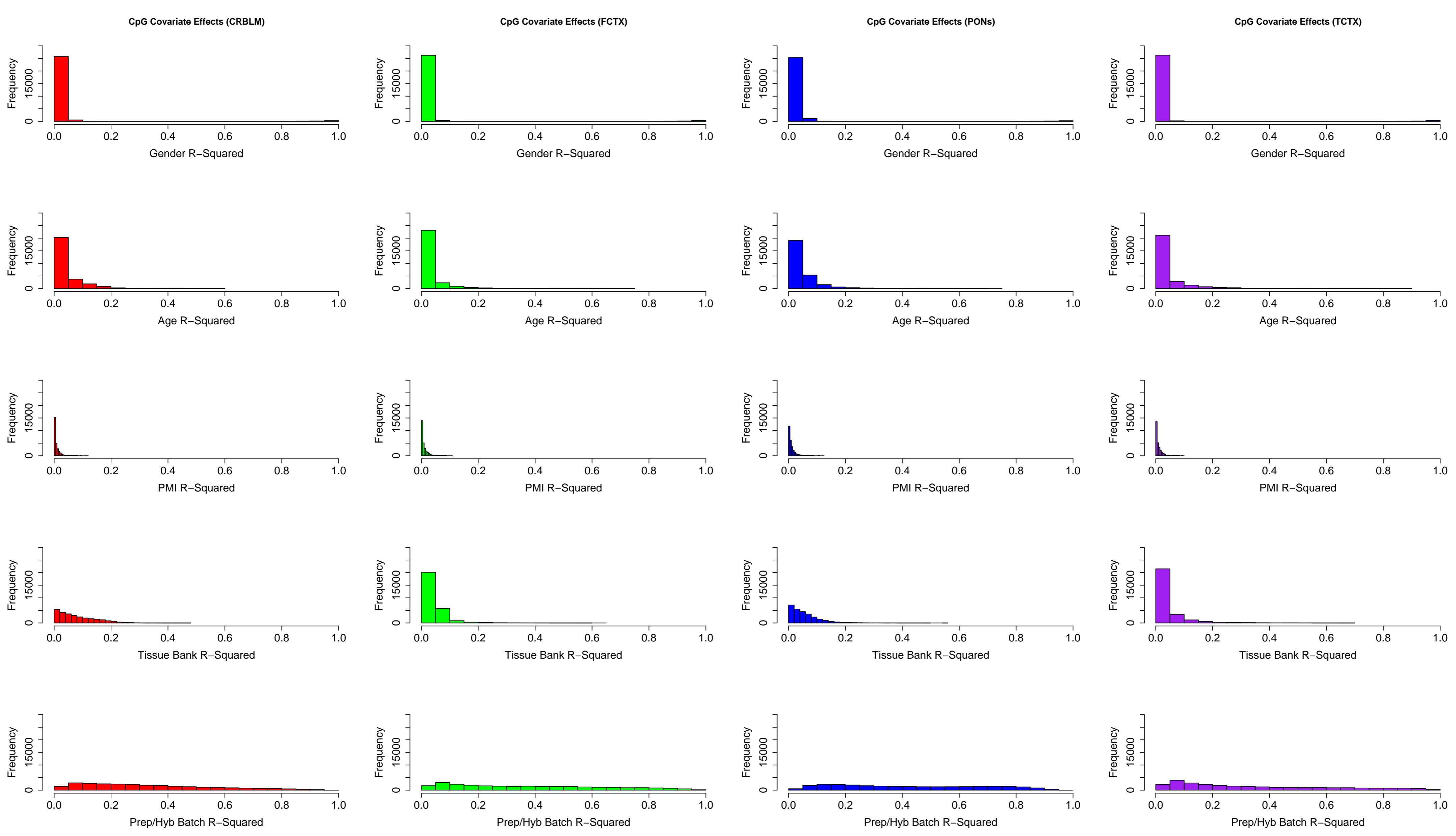

Supplement: Figure S4 — Histograms of potential covariate effects on CpG DNA methylation levels, if the data had not been adjusted for these confounds prior to QTL analysis. Each row of sub-plots represents a single covariate and each column represents a brain tissue region; CRBLM (red), FCTX (green), PONS (blue) and TCTX (purple). The histograms show the number of probes (CpG sites) on the y-axis and the R2 values from the regression of the covariates with each probe. (0.04 MB PDF) [file pgen.1000952.s004.pdf]

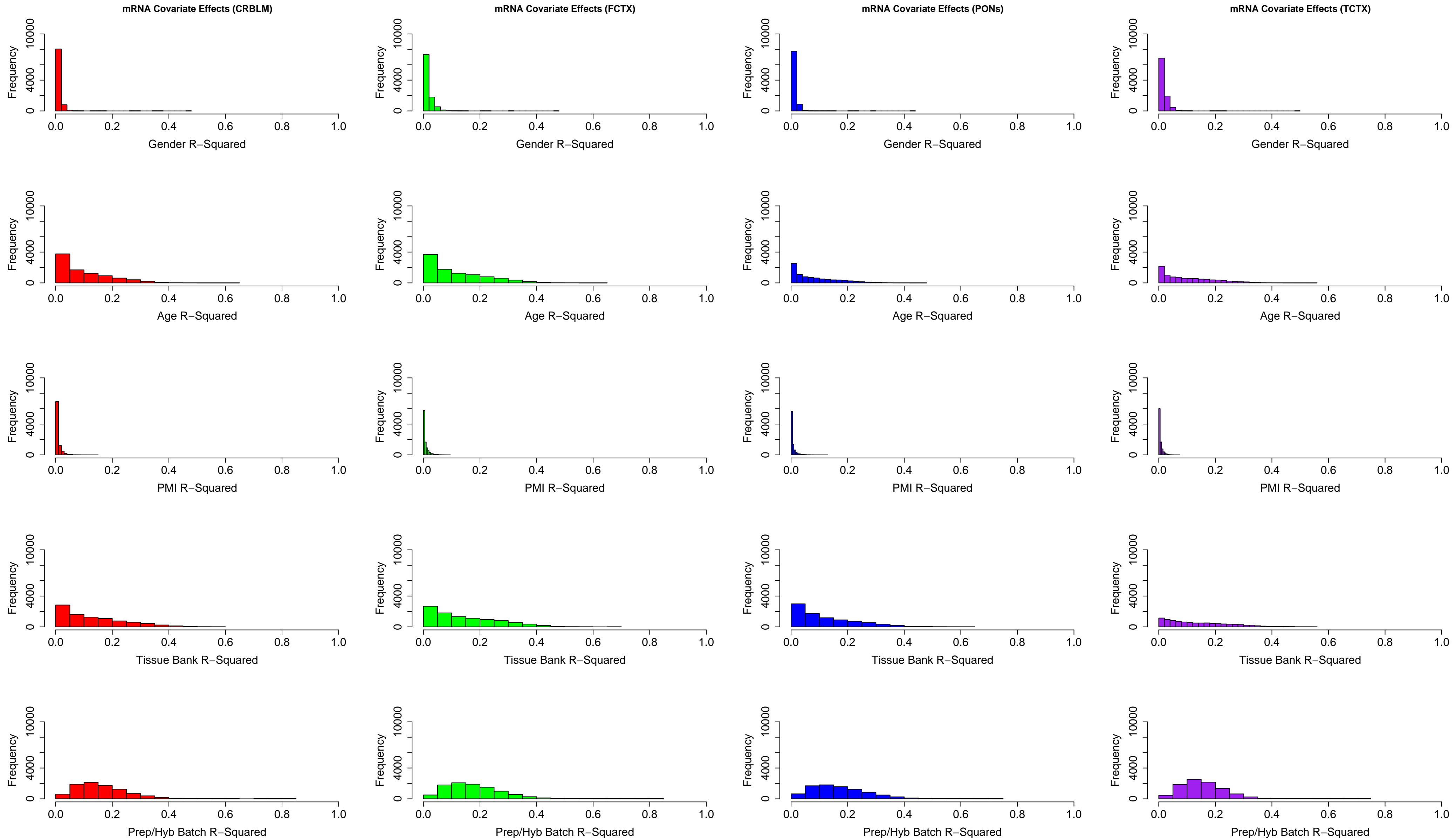

Supplement: Figure S5 — Histograms of potential covariate effects on mRNA expression levels, if the data had not been adjusted for these confounds prior to QTL analysis. Each row of sub-plots represents a single covariate and each column represents a brain tissue region; CRBLM (red), FCTX (green), PONS (blue) and TCTX (purple). The histograms show the number of probes (mRNA transcripts) on the y-axis and the R2 values from the regression of the covariates with each probe. (0.04 MB PDF) [file pgen.1000952.s005.pdf]

**CpG**

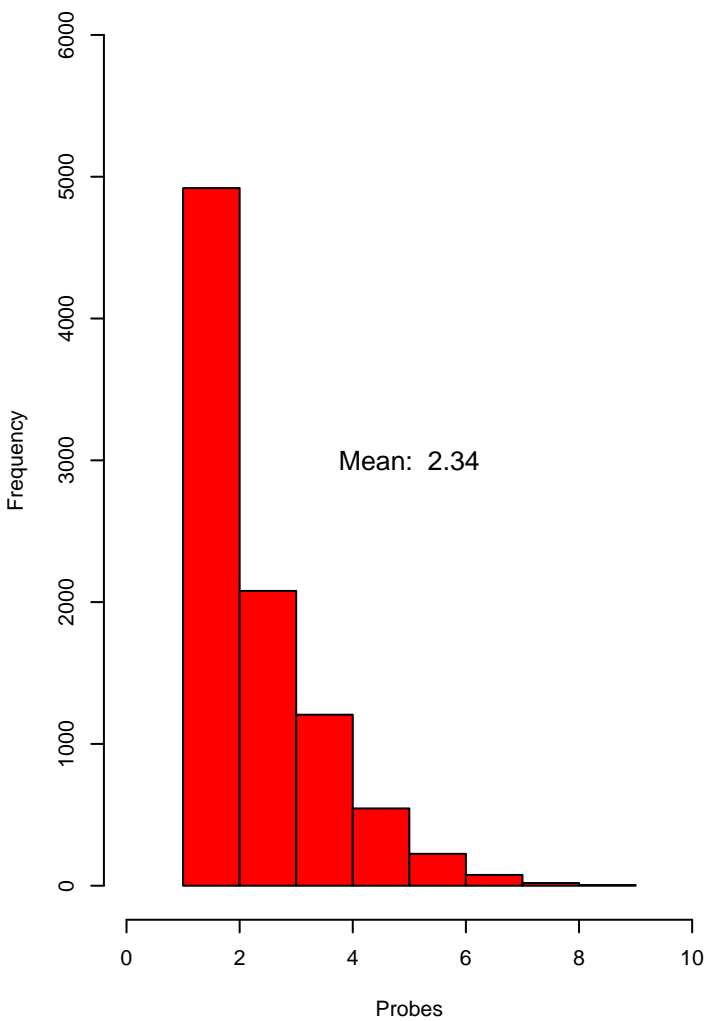

**CpG 10K resamples, 100 probes per,  $p \leq 1e-08$**

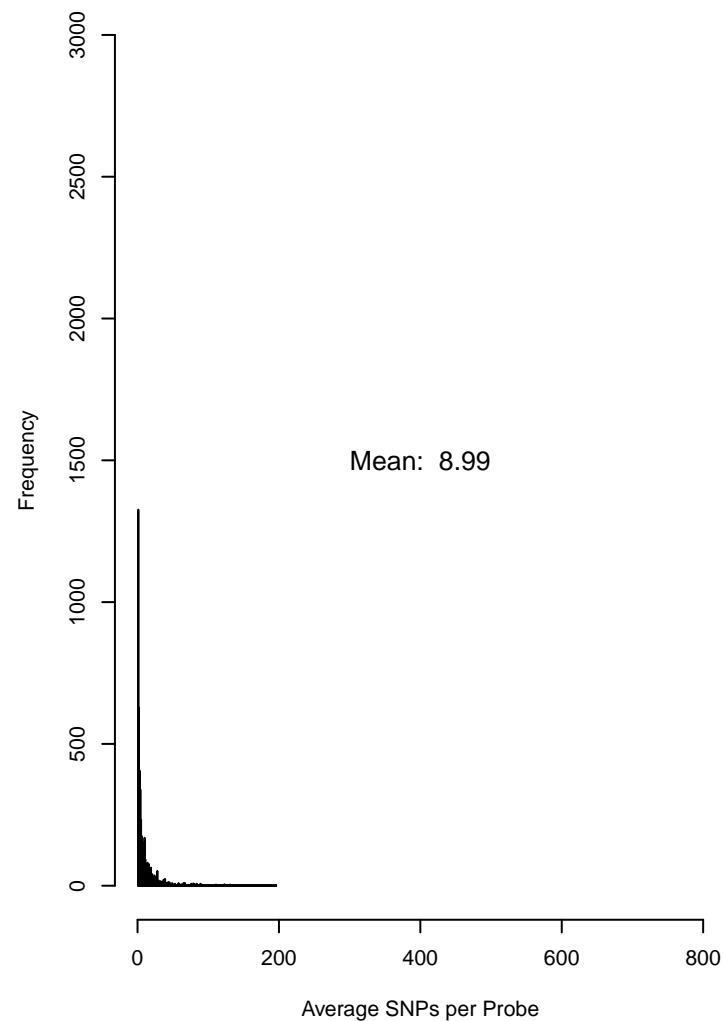

**CpG**

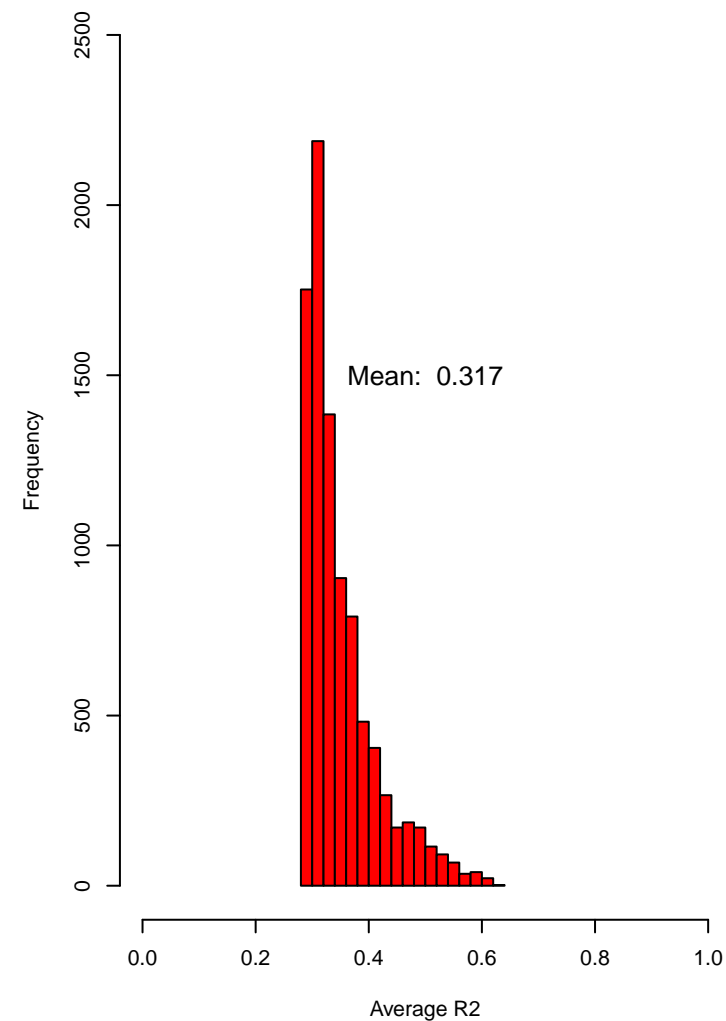

**mRNA**

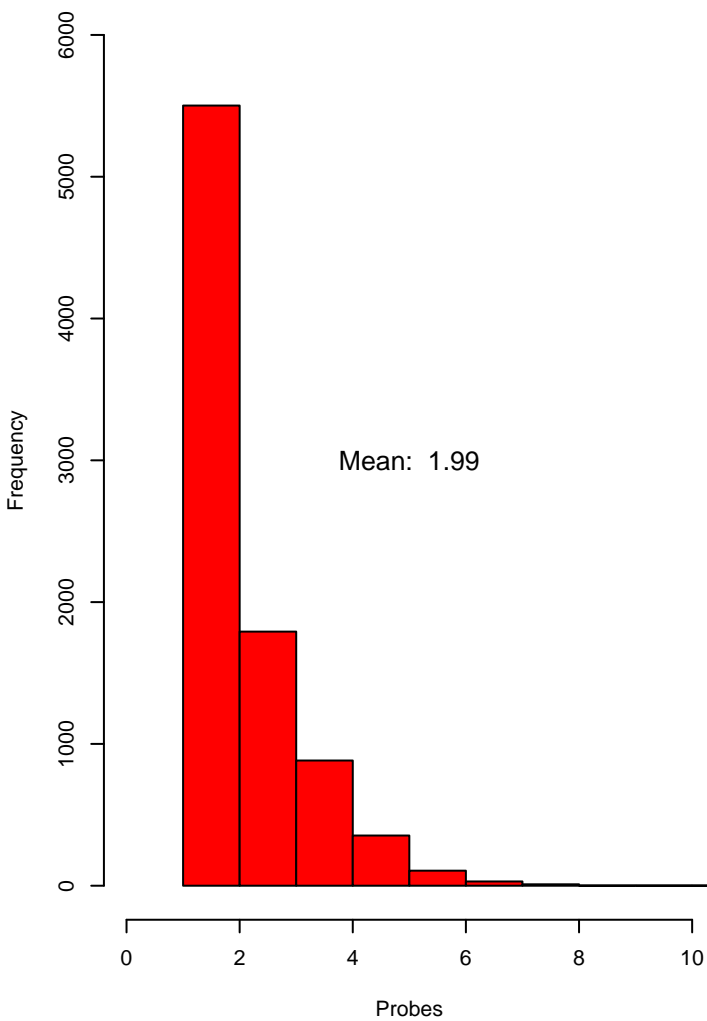

**mRNA**

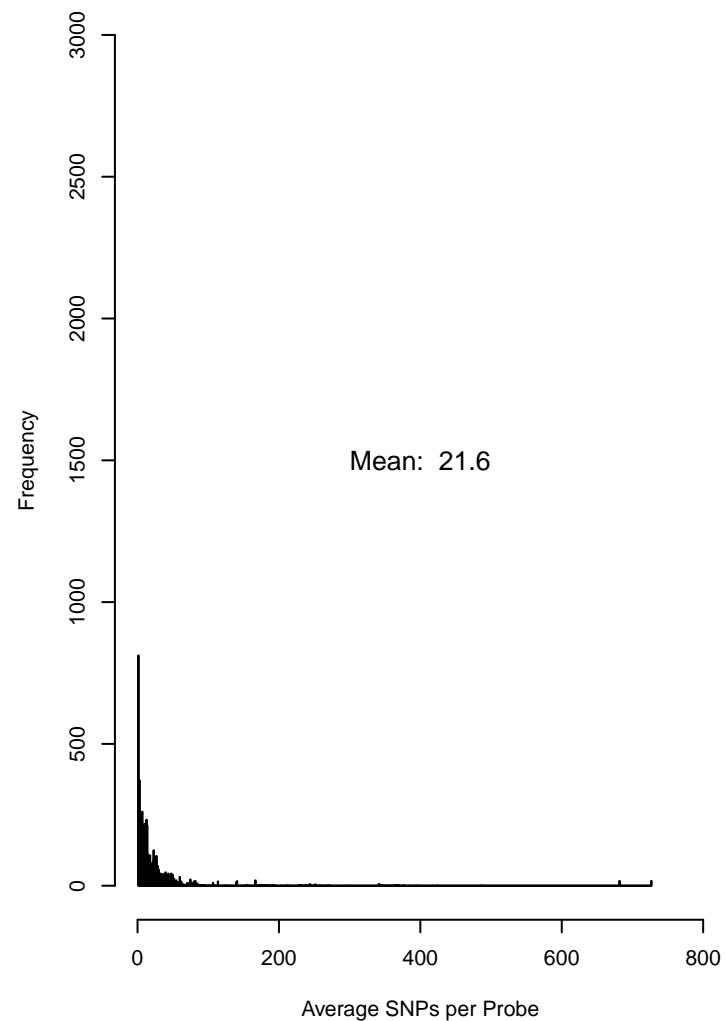

**mRNA**

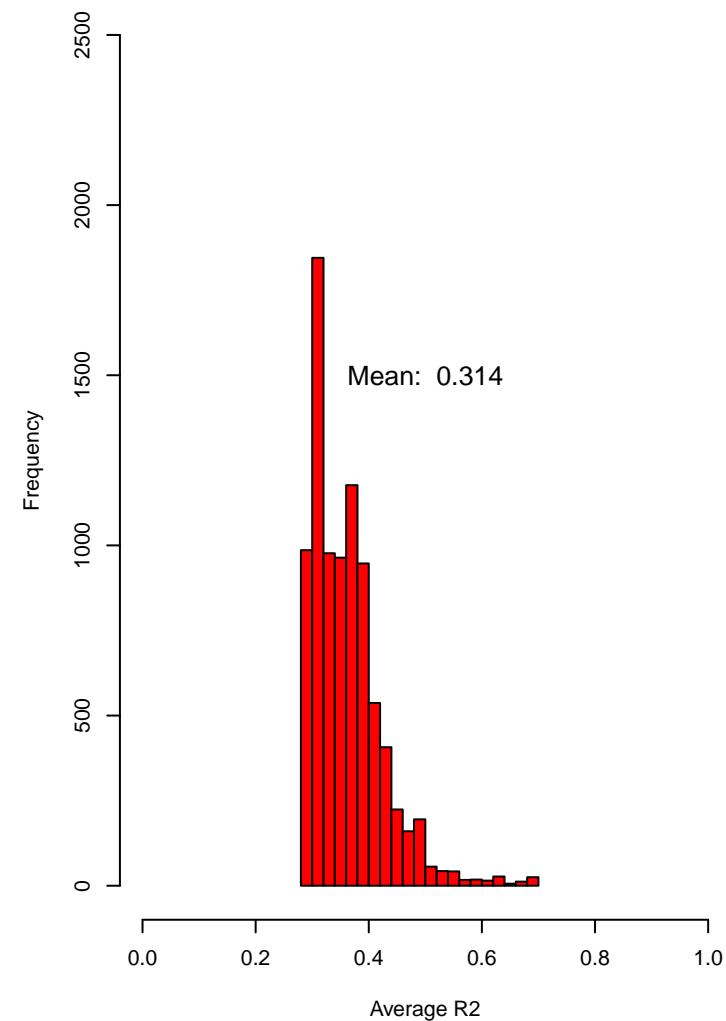

Supplement: Figure S6 — Histograms representing the results of the resampling analysis. Each row represents a different assay type (CpG and mRNA) and each column represents a different metric of the resampling; number of probes, average number of SNPs per probe and average R2 of probe/SNP correlations. (0.11 MB PDF) [file pgen.1000952.s006.pdf]

CRBLM (Region chr14:64214886–64714886)

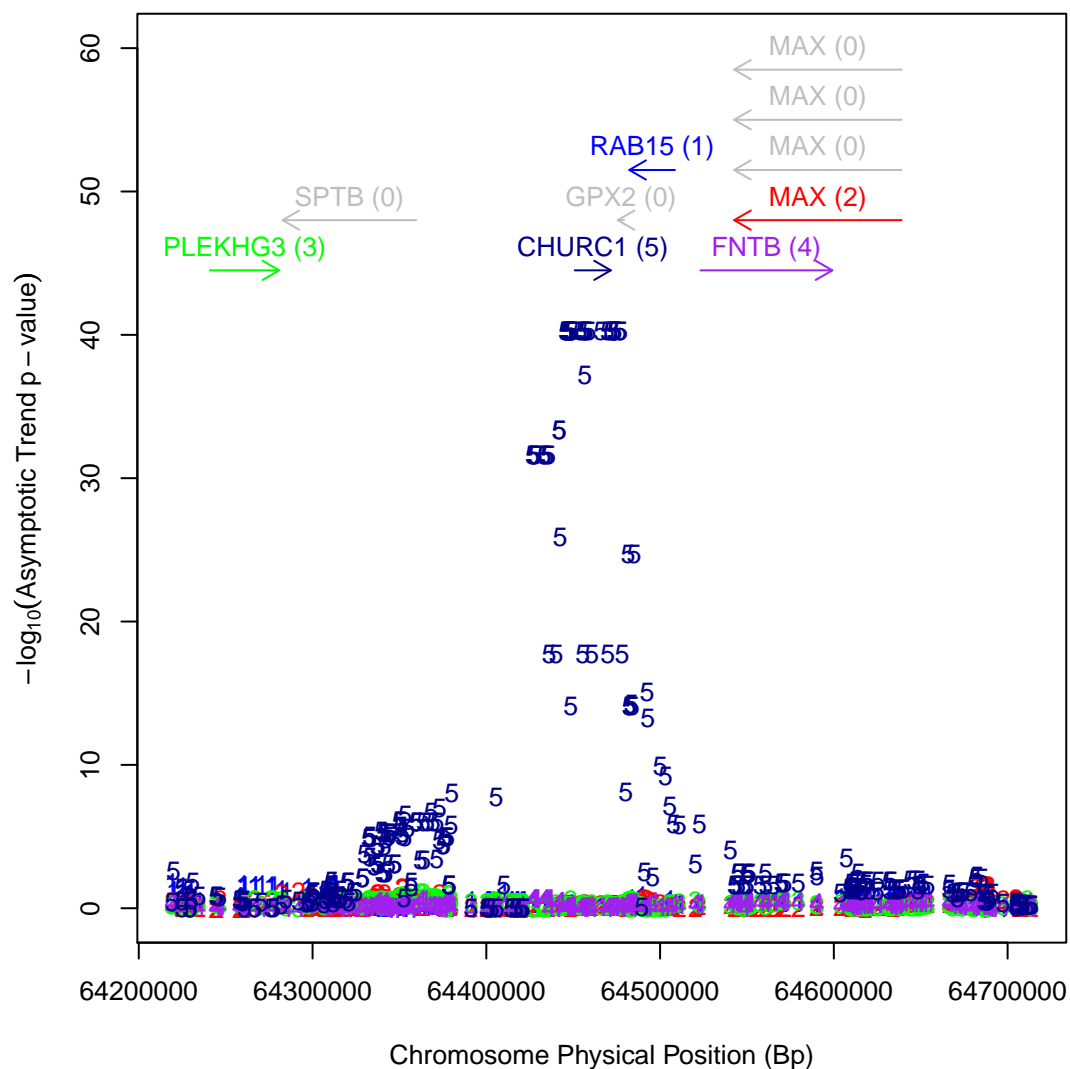

FCTX (Region chr14:64214886–64714886)

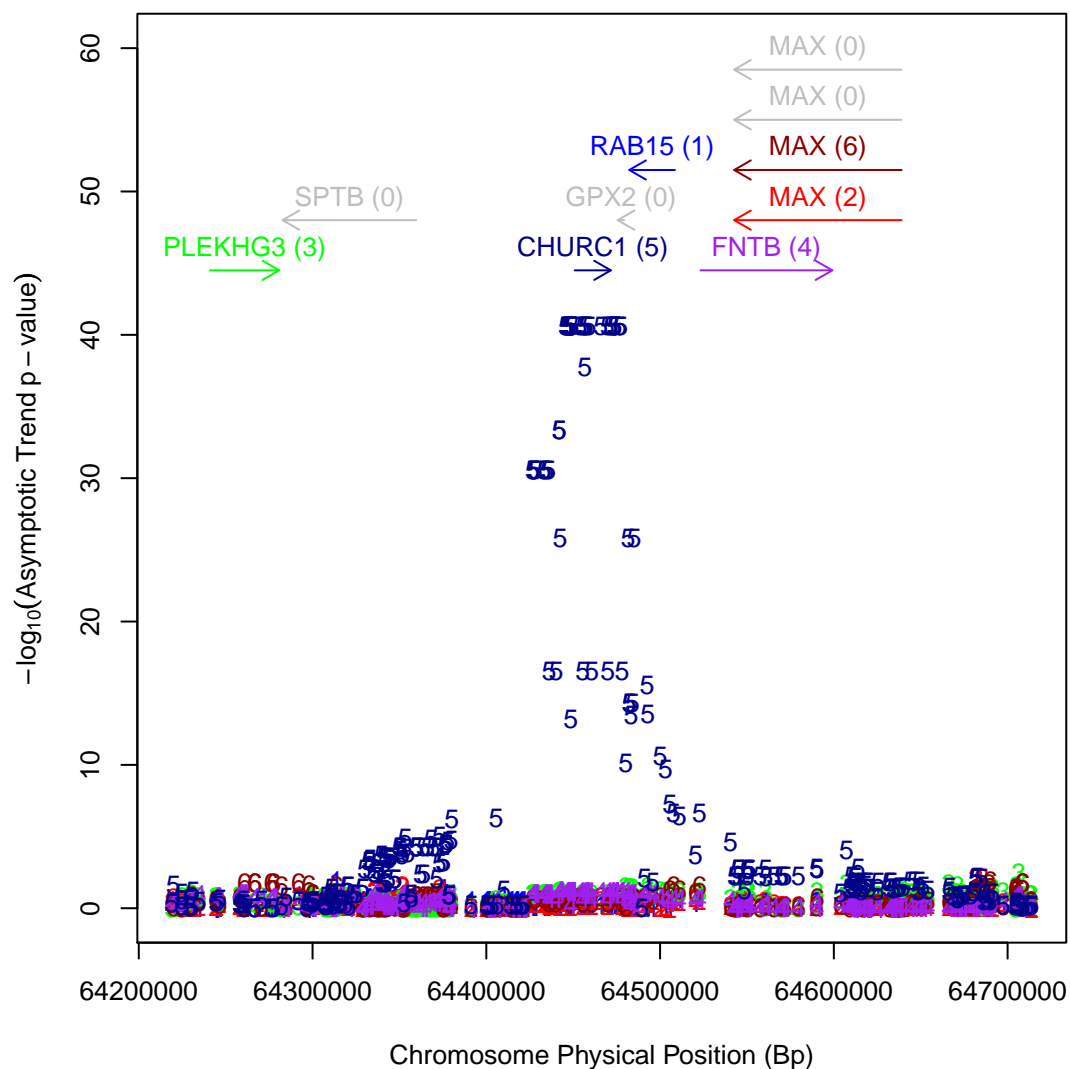

PONS (Region chr14:64214886–64714886)

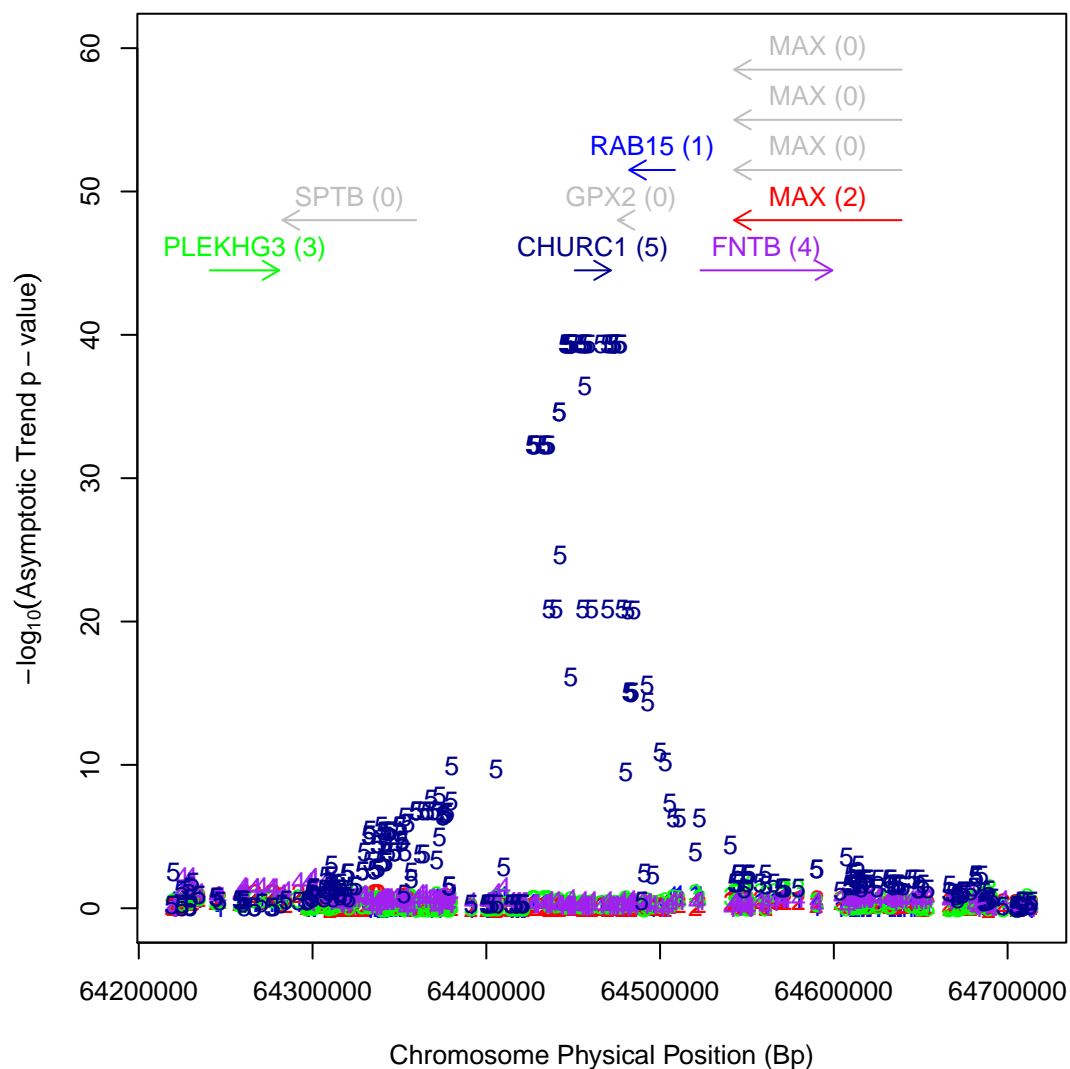

TCTX (Region chr14:64214886–64714886)

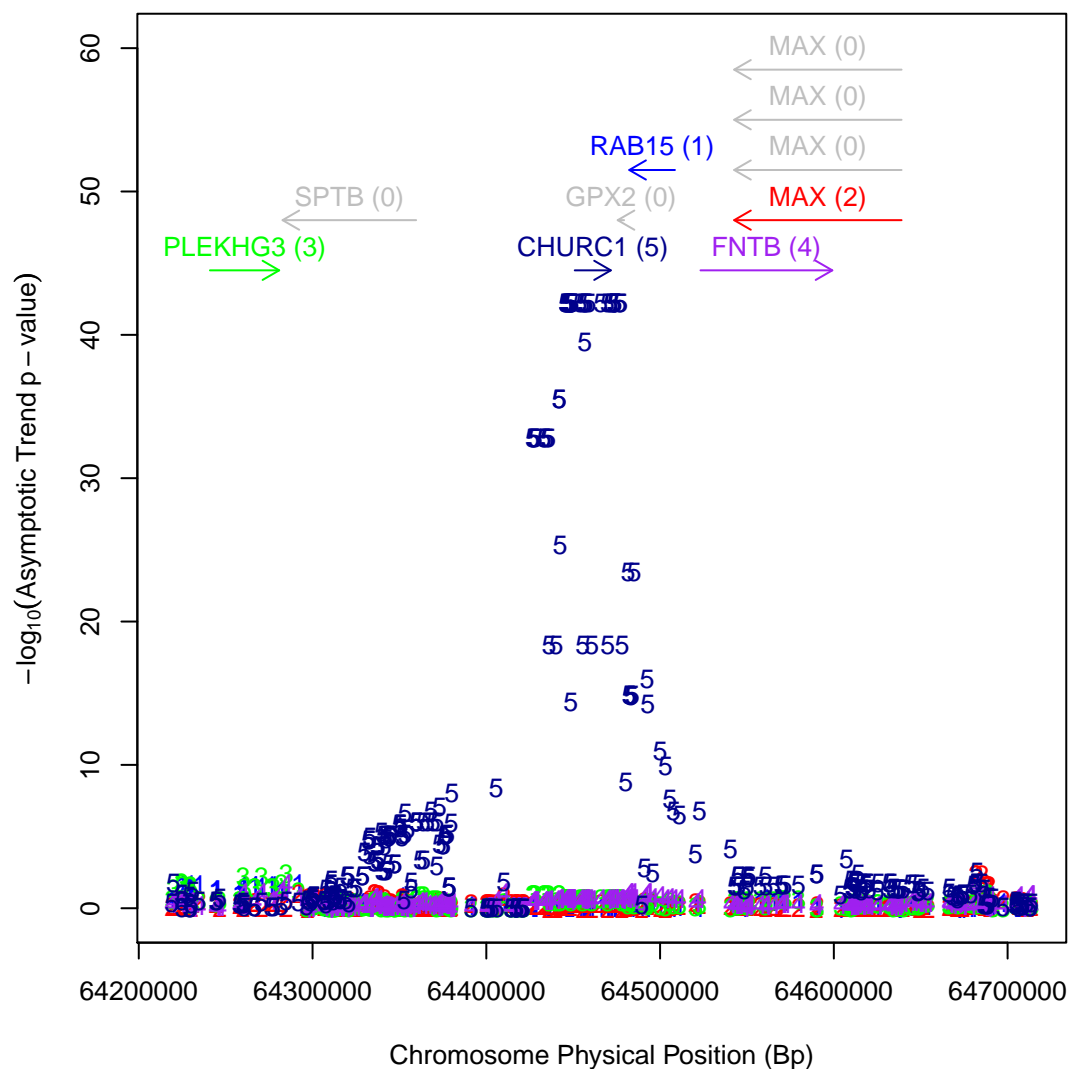

Supplement: Figure S7 — These plots, one for each brain tissue, show the p-values of correlations between SNPs and mRNA transcripts in a 500Kb region centered on CHURC1. Where a eQTL in this genomic region is present in all four brain tissues. A cis QTL for CHURC1 has also been reported within liver (Schadt et al., 2008). Within each plot the X-axis is the physical position along this region of the chromosome and the Y-axix is the −log10(asymptotic p-values) for the correlations. The p-values are colored and numbered to match the annotated transcripts labeled in the top portion of the plots. Thus in CRBLM, PONS and TCTX the dark blue ‘5’s are p-values for CHURC1 and in FCTX the dark red ‘6’s are p-values for CHURC1. mRNA transcript annotations shown in grey are those where a probe in present on the expression platform but are not detected in 95% of the tissue. (0.43 MB PDF) [file pgen.1000952.s007.pdf]

**CRBLM (Region chr10:121811339–122811339)**

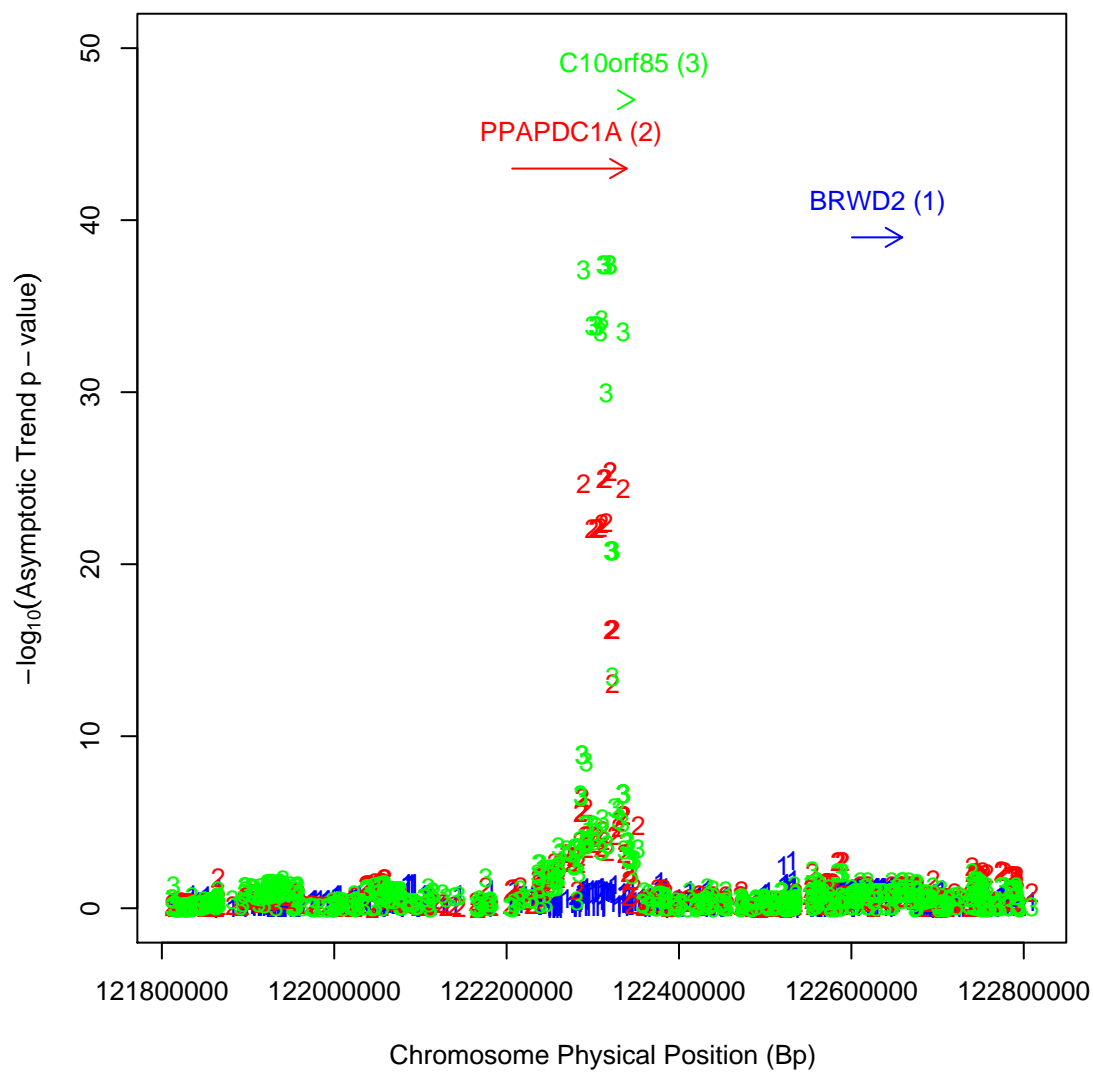

**FCTX (Region chr10:121811339–122811339)**

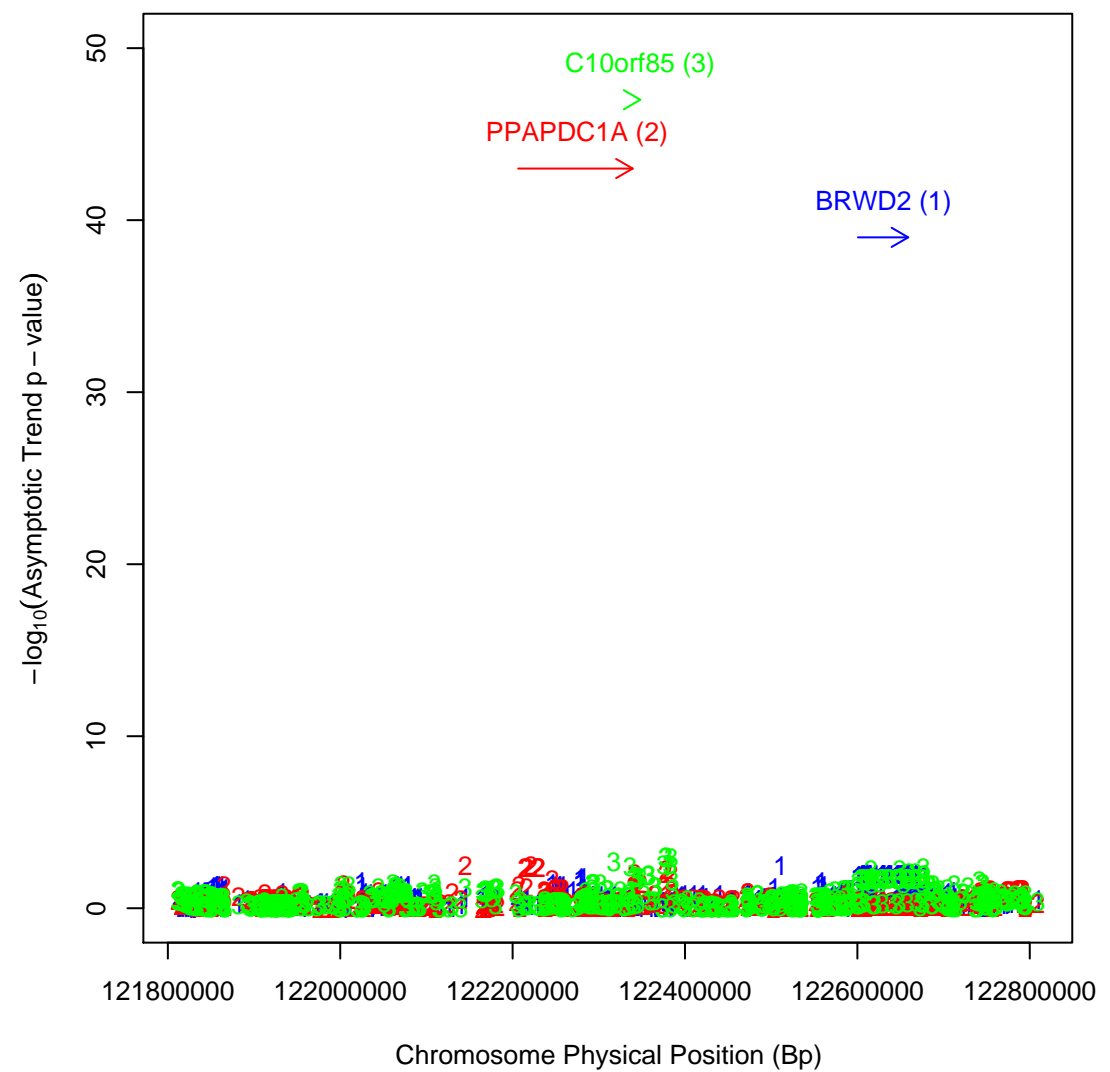

**PONS (Region chr10:121811339–122811339)**

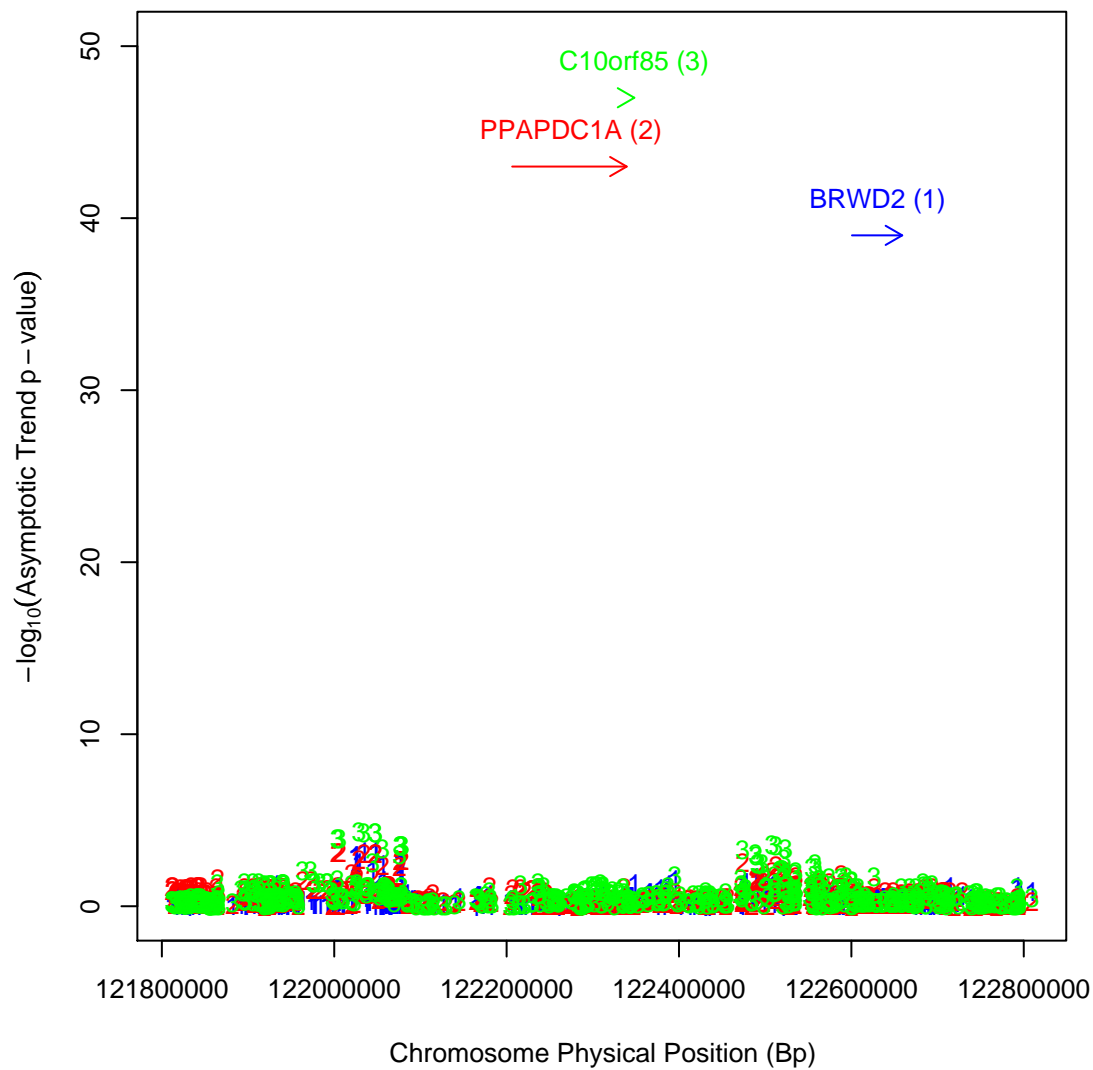

**TCTX (Region chr10:121811339–122811339)**

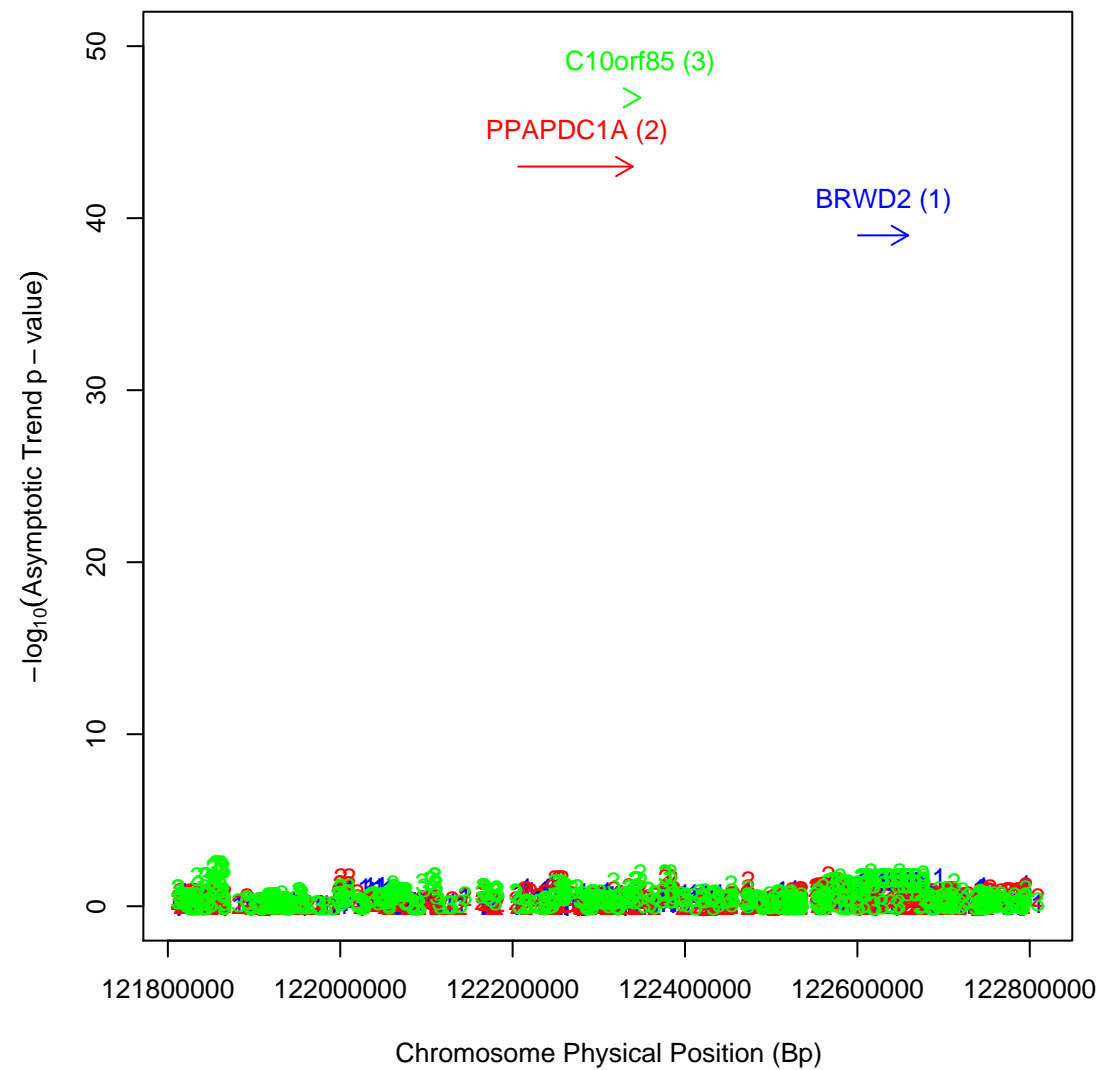

Supplement: Figure S8 — These plots show an example of a cis eQTL for an mRNA that appears to be tissue specific. The plots, one for each brain tissue, show the p-values of correlations between SNPs and mRNA transcripts in a 1Mb region centered on PPAPDC1. Within each plot the X-axis is the physical position along this region of the chromosome and the Y-axix is the −log10(asymptotic p-values) for the correlations. The p-values are colored and numbered to match the annotated transcripts labeled in the top portion of the plots. Thus in CRBLM the red ‘2’s are p-values for SNPs correlated with PPAPDC1A. Also present at this same genomic locus is another tissue specific eQTL for the mRNA transcript C10orf85, shown as green ‘3’s. (0.51 MB PDF) [file pgen.1000952.s008.pdf]

**CRBLM (N = 4708)**

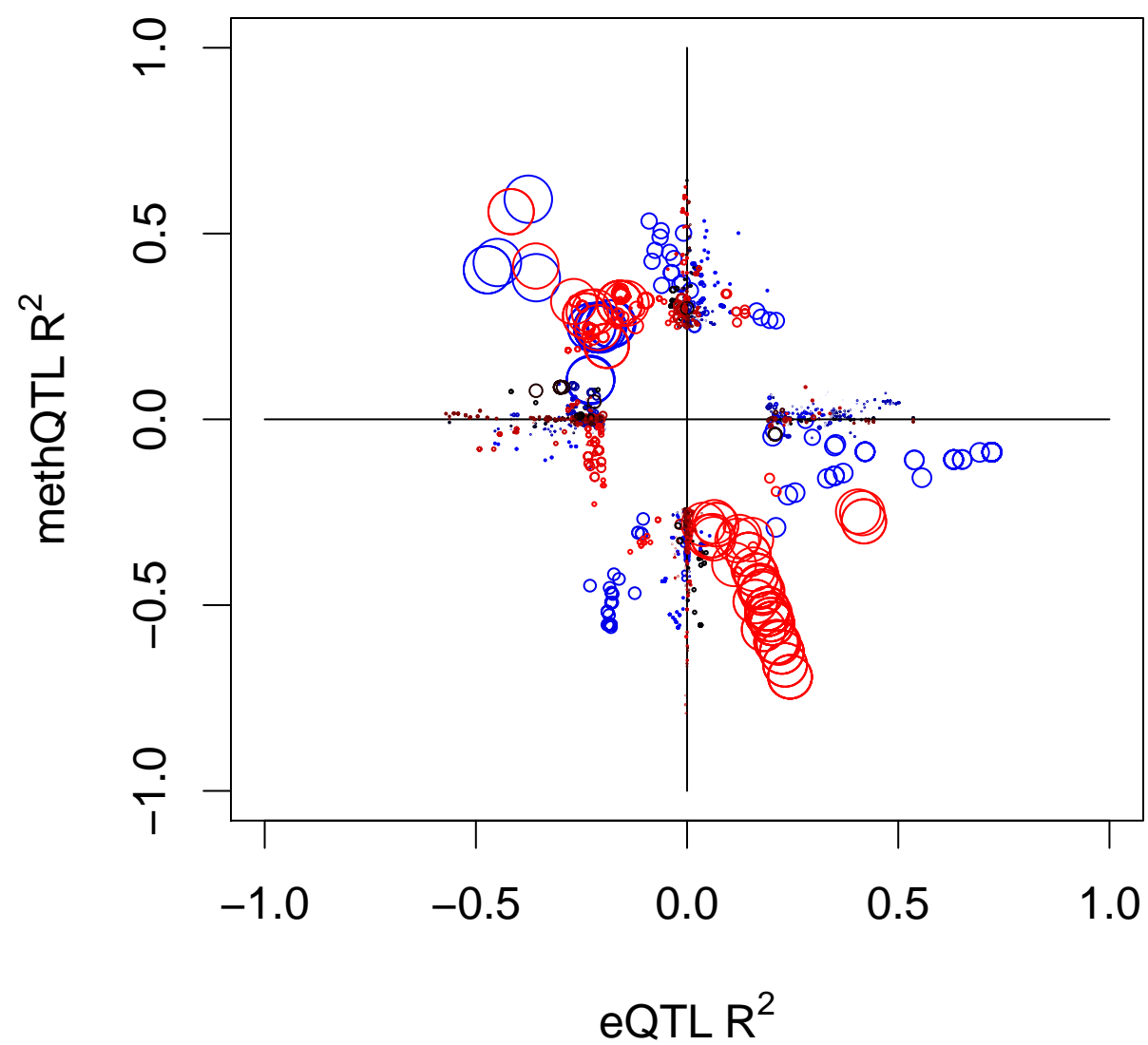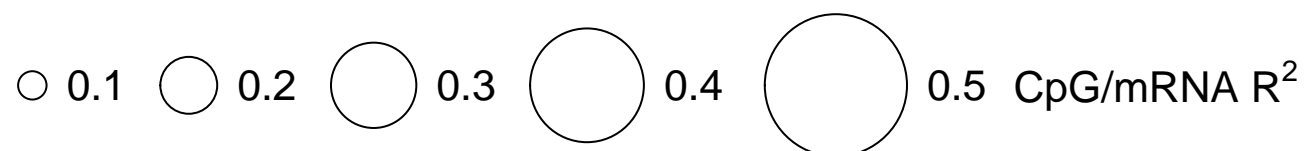

**FCTX (N = 4016)**

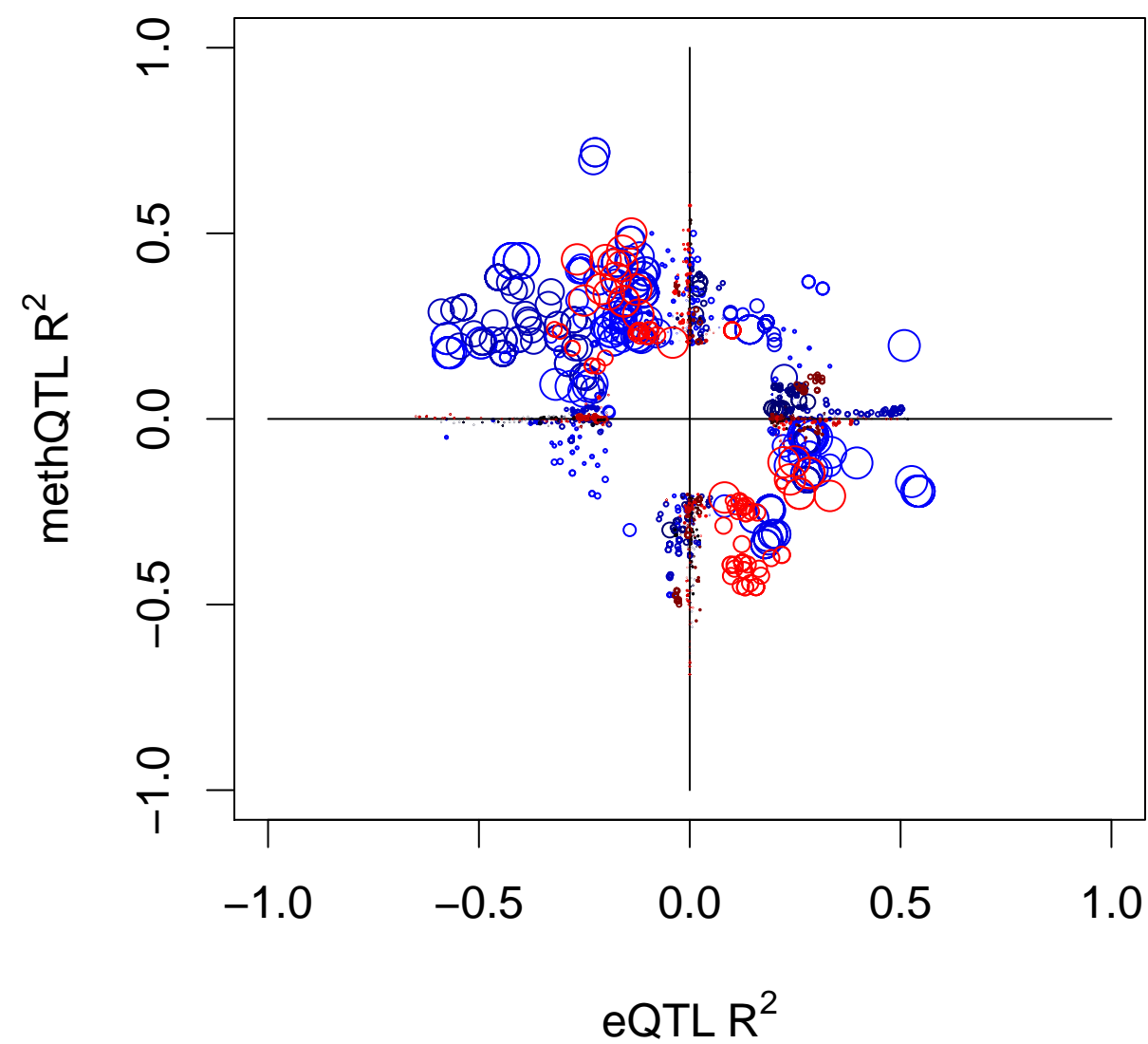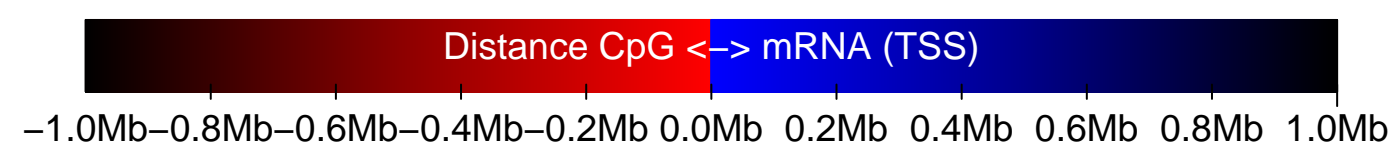

**PONS (N = 2340)**

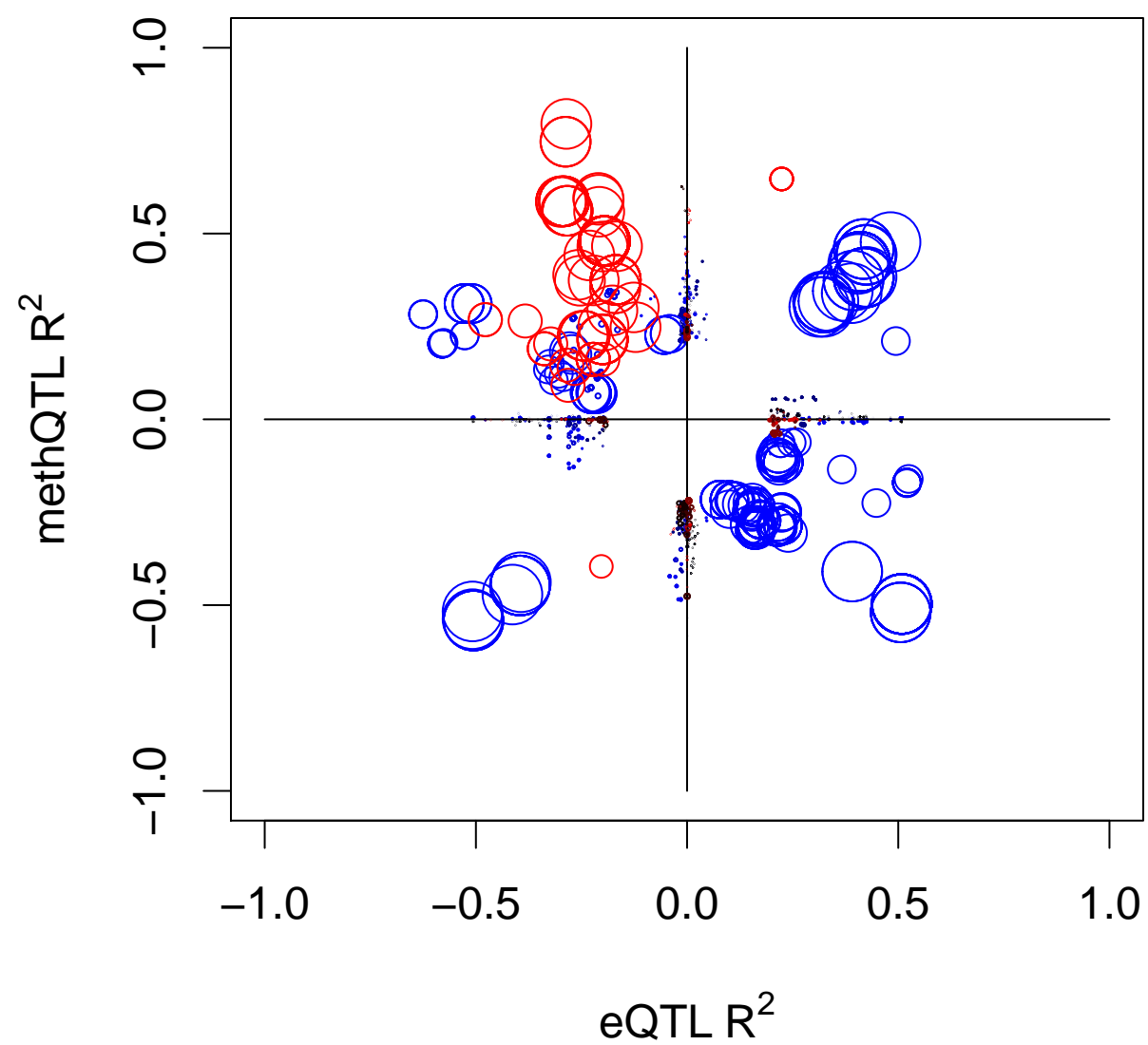

**TCTX (N = 5323)**

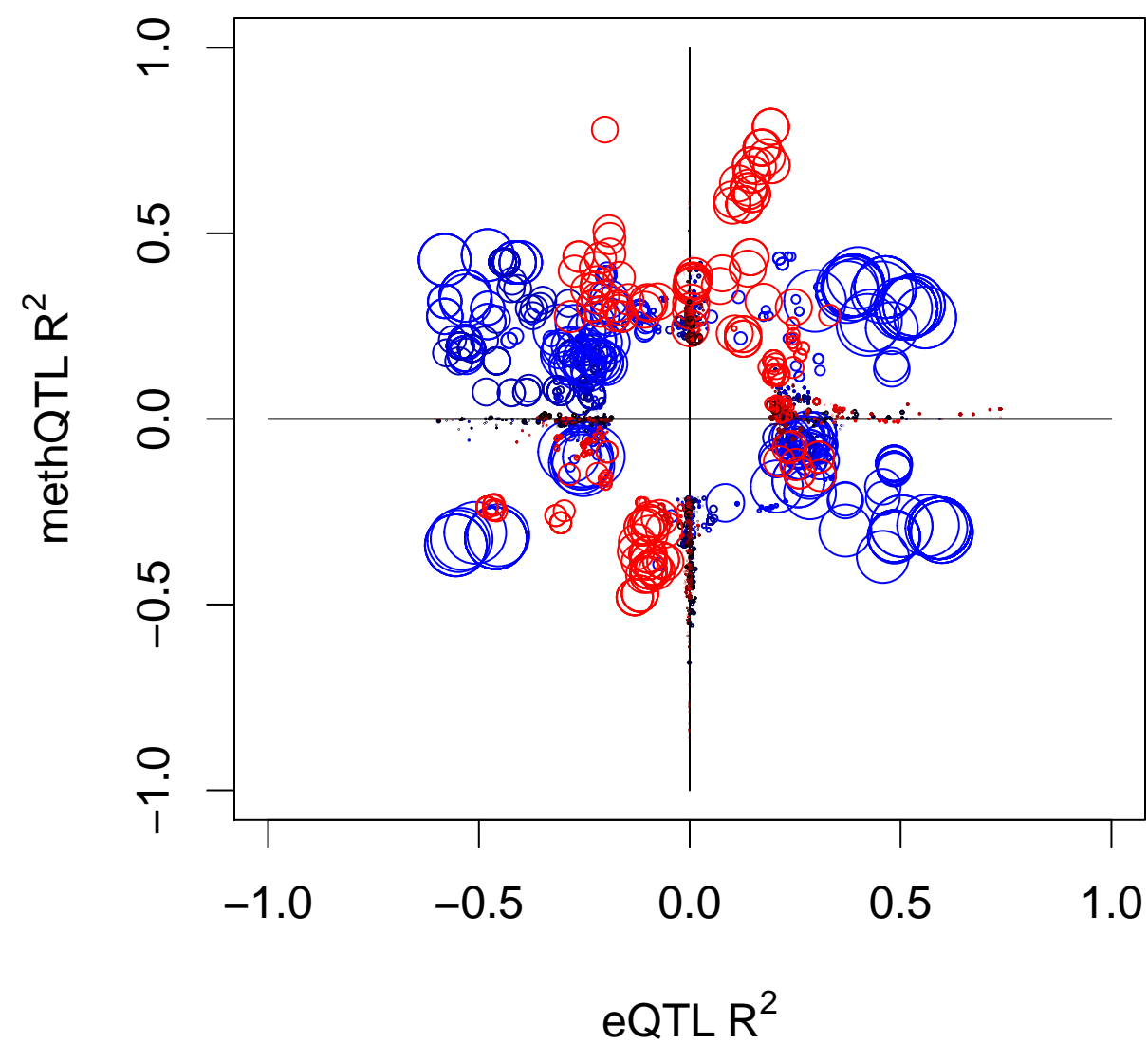

Supplement: Figure S9 — Intersection of QTLs for CpG and mRNA traits. Plots shown for each tissue region; cerebellum, frontal cortex, pons and temporal cortex. Per tissue for every pairing of CpG site and mRNA transcript where the CpG was within 1Mb of the mRNA TSS and both the CpG and mRNA have a significant cis QTL; i.e., a triplet is formed between SNP, mRNA and CpG, where the SNP was significantly correlated in cis with either the CpG or mRNA of the CpG-mRNA cis pairs under consideration. For these triplets we plotted the eQTL R2 (X axis) and the methQTL R2 (Y axis). The R2 values are shifted into one of four quadrants (without changing the effect size) based on the positive and negative combinations of correlations for the eQTLs and methQTLs. A positive correlation with mRNA means that the level of expression is increased with the minor allele of the SNP and a negative correlation means that the mRNA expression level is decreased with the minor allele of the SNP. For CpGs a positive correlation implies that the level of methylation is increased at a CpG site with the minor allele and a negative correlation implies that the level of methylation is decreased with the minor allele. The top-left quadrant contains negative eQTL and positive methQTL correlations. The top-right quadrant contains contains eQTLs and methQTLs where the correlation was positive in both. The bottom-left quadrant contains eQTLs and methQTLs where the correlation was negative in both. The bottom-right quadrant contains positive eQTL and negative methQTL correlations. The triplets are plotted as circles where the radius of the circle represent the R2 value between the CpG site and mRNA transcript. The color of circle represents the distance between the CpG site and the mRNA TSS, where red indicates that the CpG site is closer upstream to the mRNA TSS; blue indicates that the CpG site is close the the mRNA TSS but downstream; and black indicates that the CpG site is farther from the mRNA TSS both up or downstream. Tr [file pgen.1000952.s009.pdf]
