# Supplementary material for: Abundant Quantitative Trait Loci Exist for DNA Methylation and Gene Expression in Human Brain
Source: PLoS Genet. 2010 May 13;6(5):e1000952. doi: 10.1371/journal.pgen.1000952 (PMC2869317; doi:10.1371/journal.pgen.1000952)
Supplement: Text S1 — Genetic control of DNA methylation and expression across human brain tissues. (0.06 MB DOC) [file pgen.1000952.s014.doc]

**Supplemental Methods and Materials**

**ABUNDANT QUANTITATIVE TRAIT LOCI EXIST FOR DNA METHYLATION AND GENE EXPRESSION IN HUMAN BRAIN**

**Section 1 - Samples**

**1.1 Subject Characteristics**

Frozen tissue samples of the frontal cortex, temporal cortex, cerebellum and caudal pons were obtained from each of 150 subjects who had donated their brains for medical research. One hundred and fourteen brains were sampled from the University of Maryland Brain Bank, Baltimore (<http://medschool.umaryland.edu/BTBank/>). Thirty-six brains were sampled from the Department of Neuropathology, Johns Hopkins University, Baltimore, either as routine autopsy cases (n = 10), or as part of the National Institute on Aging-sponsored Baltimore Longitudinal Study of Aging (BLSA, n = 26; <http://www.grc.nia.nih.gov/branches/blsa/blsanew.htm>). All individuals were of non-Hispanic, Caucasian ethnicity, and none had a clinical history of neurological or cerebrovascular disease, or a diagnosis of cognitive impairment during life.

Demographics, tissue source and cause of death for each subject are shown in Table S1. Average age at time of death was 45.8 years of age (range, 15 - 101 years). One hundred and three brains (68.7%) were from male cadavers. The average post-mortem interval was 14.3 hours (range, 3 - 28 hours, missing data for 1 sample). The most common cause of death was accidental injury (n = 55 cases), followed by cardiovascular disease (n = 31), drug intoxication (n = 12), and pulmonary embolism (n = 3). Other causes of death included drowning (n = 3), respiratory disease (n = 2), compressional asphyxia (n = 1), suicide by hanging (n = 1), choking (n = 1), lightning strike (n = 1), liver disease (n = 1), mitral valve prolapse (n = 1), myocarditis (n = 1) and diabetic coma (n = 1). Cause of death was not available for the remaining 36 autopsies.

**1.2 Sample Preparation**

For each of the six hundred samples (150 brains x four regions), approximately 5 grams of frozen tissue was sub-dissected at either the University of Maryland Brain Bank or at the Department of Neuropathology, Johns Hopkins University, and sent on dry ice to the Laboratory of Neurogenetics (LNG), NIA. At LNG, 100-200mg aliquots of frozen tissue were sub-dissected from each sample by a neurologist (BJT, S-LL) using a fresh blade for each sample. Samples were kept on dry ice to avoid thawing. Separate pieces were cut for DNA extraction to be used in SNP genotyping assays, RNA extraction for expression assays and DNA extraction for methylation assays. Each tissue aliquot was stored at -80°C until use.

Genomic DNA extraction for genotyping was performed using the DNeasy Blood and Tissue Kit as per the manufacturer’s instructions (Qiagen Inc., Valencia, CA). Genomic DNA for the Infinium methylation assays was extracted using phenol-chloroform and ethanol precipitation. DNA concentration was determined using a Nanodrop ND-1000 spectrophotometer (Thermo Scientific, Wilmington, DE), and DNA extraction was repeated using a new tissue aliquot for samples with DNA concentration less than 50ng/ul, or with a 260/280 ratio less than 1.7.

For each of the 600 brain samples, total RNA was prepared from approximately 100mg of tissue using a glass-Teflon homogenizer and 1mL TRIzol (Invitrogen, Carlsbad, CA) according to the manufacturer’s instructions. RNA samples were resuspended in RNAse free water to a final concentration of >500ng/uL. In each case, the same RNA preparation was used for mRNA analysis.

**Section 2 – Assay methodology**

**2.1 SNP genotyping**

SNP genotyping was performed using DNA extracted from cerebellar tissue for each subject using Infinium HumanHap550 version 3 BeadChips (Illumina Inc., San Diego, CA) according to the manufacturer’s instructions. Genotype data was analyzed using the Genotyping Analysis Module 3.2.32 within the BeadStudio software version 3.1.4 (Illumina Inc.).

**2.2 RNA Expression**

Profiling of 22,184 mRNA transcripts was performed using HumanRef-8 version 2 Expression BeadChips (Illumina Inc.) in accordance with the manufacturer’s instructions and as previously described (van der Brug et al., 2008). mRNA expression data were analyzed using the Gene Expression Module 3.2.7 within BeadStudio.

**2.3 CpG Methylation**

Genome-wide methylation profiles were generated using Infinium HumanMethylation27 BeadChips (Illumina Inc.), which measure DNA methylation at 27,578 CpG dinucleotides spanning 14,495 genes. A GenePaint automatic slide processor robotic system was used to simultaneously process twenty-four BeadChips. Briefly, 1ug of genomic DNA underwent bisulfite conversion using the WZ-96 DNA Methylation Kit according to the manufacturer’s protocol (Zymo Research Corp, Orange, CA). Incubation conditions used during conversion were as follows: (95°C for 30 seconds, 50°C for 1 hour) for 16 cycles, hold overnight at 4°C. Unmethylated cytosines were chemically deaminated to uracil in the presence of bisulfite, while methylated cytosines were refractory to the effects of bisulfite and remained as cytosines. Methylation was then detected as a C/T nucleotide polymorphism at each CpG site. After bisulfite conversion, each sample was whole-genome amplified, fragmented, and hybridized to the BeadChip. DNA molecules anneal to locus-specific DNA oligomers. Two bead types correspond to each CpG locus, one to the methylated (C) state, and the other to the unmethylated (T) state. After extension, the BeadChip was fluorescently stained, and scanned using a BeadArray laser confocal scanner to measure the intensities of unmethylated and methylated bead types for each locus. Data were analyzed using the Methylation Analysis Module 3.2.0 within BeadStudio. Intensities were not normalized. The DNA methylation value for each locus is reported by BeadStudio as AVG_Beta, which is a continuous variable between 0 and 1 representing the ratio of the intensity of the methylated bead type to the combined locus intensity.

**Section 3 – Data analysis**

**3.1 Genotyping data**

The threshold call rate for inclusion of the sample in analysis was 95%. Two samples initially had a call rate below this threshold, but were successfully re-genotyped using fresh DNA aliquots. Thus all 150 brain samples had a call rate greater than 95%, and were included in the subsequent analyses (average call rate = 99.86%; range 97.72% - 99.95%, based on the *missing* procedure within the PLINK v1.04 software toolset (Purcell et al., 2007).

The gender of the samples reported to LNG by the brain banks was compared against their genotypic gender using PLINK 's *check-sex* algorithm, which determines a sample’s genotypic gender based on heterozygosity across the X chromosome. Two samples with gender discrepancies were detected. One of these arose from a clerical error at the brain bank and was included in the analysis after correction of the clinical information, whereas the other sample (UMARY1496) was removed from subsequent analysis.

To confirm the ethnicity of the samples, Identity-By-State (IBS) clustering and multidimensional scaling analyses were performed within PLINK using the genotypes from the brain samples that had been merged with data from the four HapMap (HapMap Consortium 2005) populations (n = 32 Caucasian (CEU), 12 Han Chinese, 16 Japanese and 24 Yoruban non-trio samples previously genotyped by Illumina and assayed on the Infinium HumanHap500 version genotyping chips). Two samples were outliers based on population and excluded from further analysis (UMARY4545, UMARY927). Results are shown in Figure S1.

Genotype data of the samples were compared for cryptic relatedness using the Identity-By-Descent (IBD) procedure within PLINK. No samples were found to be from related individuals.

Mach software version 1.0.16 (Li and Abecasis, 2006) and HapMap CEU phase data (release 22) were used to impute genotypes for ~2.5 million SNPs. Imputed SNPs were excluded if the linkage disequilibrium r^2^ values between imputed and known genotypes was less than 0.3, and if their posterior probability averages were less than 0.8 for the most likely imputed genotype. For each of the four tissue regions, SNPs were also excluded if: (a) call rate was less than 95%, (b) Hardy-Weinberg equilibrium (HWE) p-value was less than 0.001, and (c) the SNP had less than 3 minor homozygotes present.

**3.2 – RNA expression data**

Raw intensity values for each probe were transformed using the rank invariant normalization method (Workman 2002, Schadt 2001 and Tseng 2001) ) and then log_2_ transformed for mRNA analysis. The following mRNA samples were excluded based on this based on low overall detection of probes and/or expression levels; UMARY1668 (CRBLM), UMARY1909 (FCTX), UMARY4543 (PONS) and UMARY4782 (PONS). The following individuals were not run on mRNA expression arrays for any tissue region; BLSA1672, JHU1344 and JHU1361. Only probes that were detected in 95% of all samples within a tissue type were used for further analysis. In total 10326 mRNA transcripts were analyzed within at least one brain tissue region; 8076 mRNA transcripts were analyzed within all four brain tissue regions. Shared detection rates are shown as Venn diagrams in Figure S2.

**3.3 – CpG methylation data**

The threshold call rate for inclusion of samples in the analysis was 95%. Based on this metric, 9 cerebellar samples (UMARY933, UMARY1465, UMARY4593, UMARY4640, UMARY4726, UMARY4727, UMARY4842, JHU1361, and BLSA1640), 6 pons samples (UMARY384, UMARY1541, UMARY1583, UMARY1613, UMARY1907 and UMARY4903), 2 frontal cortex samples (UMARY1712, BLSA1840) and 8 temporal cortex samples (UMARY1865, UMARY1866, UMARY1867, UMARY4545, UMARY4598, UMARY4726, UMARY4727 and UMARY5087) were excluded from analysis. The remaining brain samples had an average detection rate of 99.84% (range 95.0% to 99.98%).

The gender of the samples reported to LNG by the brain banks were compared against their assayed gender based on values of methylation from CpG sites on the X chromosome (Bibikova 2006 and Carrel 205). Four samples with gender discrepancies were detected and were removed from subsequent analysis (BLSA2102-crblm, UMARY1862-crblm, UMARY880-pons, UMARY4540-tctx). The resulting HCL sample tree based on Chromosome X sites, after removal of these four individuals is shown in Figure S3.

For each tissue region, CpG sites were excluded from analysis if they were detected in less than 95% of samples.

**3.4 - Clustering of Samples by Brain Region**

Performing a Hierarchical Clustering (HCL) (Eisen 1998) of the sample profiles using the TM4 MeV version 4.1.01 tool (Saeed 2003), with Euclidian distances and ‘Average Linkage clustering’ resulted in the samples separating fairly well by brain tissue region. Where in mRNA separation of samples into 4 clusters matching brain tissue region was clear. The frontal and temporal cortices could not be separated in within CpG data. For clustering all detected data was used for mRNA; only detected data for autosomal probes was used in clustering of the CpG data, otherwise sub-clusters based on gender appeared. The HCL samples trees were saved a Newick tree files and plotted again using the HyperTree tool (http://hypertree.sourceforge.net/).

**3.5 - Correction for known Biological and Methodological Covariates**

Prior to quantitative trait loci analysis each trait was adjusted using the available biological and methodological covariates in an attempt to remove the influence of these potentially confounding affects. In R each trait was regressed using the following model:

Y=β_0_ + β_1_X_1_+… β_n_X_n_ + ε

Where Y is the trait profile (log_2_ normalized mRNA expression intensities and raw values for CpG DNA methylation) and X_1_ … X_n_ represent the biological covariates Age and Gender and the methodological covariates post mortem interval (PMI), which Brain Bank the samples was from and which preparation / hybridization batch the sample was processed in. Within this model gender, tissue bank and batch where treated as categorical covariates. After fitting each trait to the model the residuals from the model are kept and represent the trait in following analyses. Thus variance attributable to gender, age, post-mortem interval, tissue source and hybridization batch are removed prior to QTL analysis. These covariate data are available in Table S1. Histograms showing the proportion of traits that are potentially impacted by these covariates are shown in Figure S4 (CpG) and Figure S5 (mRNA).

**3.6 – Quantitative trait loci analysis**

For each of the four brain regions, a regression analysis was performed on the residuals described in the preceding section for (a) mRNA expression and (b) the methylation values generated for CpG sites. The trait residuals were then used as the quantitative phenotype for that probe in genome-wide association analysis looking for quantitative trait loci. These analyses were performed using the *assoc* function within PLINK, which correlates allele dosage with change in the trait. Each of the four tissue regions was analyzed separately, and independent genome-wide association analyses were performed looking for (a) expression quantitative trait loci (eQTLs) for mRNA and (b) methylation quantitative trait loci (methQTLs) for CpG sites. The Plink toolset quantitative trait association analysis fits data to the following model:

Y=β_0_ + β_1_ADD+ ε

Where Y is the quantitative trait and ADD represent genotypes encoded as allele dosage. See Plink Quantitative trait association and Linear and Logistic models documentation for more information.

**3.7 - Correction for multiple tests**

To correct for the large number of SNPs tested per trait, a genome-wide empirical p-value was computed for the asymptotic p-value for each SNP by using 1,000 permutations of swapping sample labels of the traits, using the maxT permutation functionality provided within Plink. A permutation based method using label swapping of the traits is an appropriate method of test correction (Churchill 1994) for these analysis as it is not dependent on these quantitative traits having a normal distribution and also allows the linkage disequilibrium of the genomic regions being tested against the traits to be maintained.

To correct for the number of traits being tested per tissue region, a false discovery rate (FDR) threshold was determined based on the empirical p-values using the *fwer2fdr* function of the multtest package in R version 2.6.1. Empirical p-values were allowed to exceed this threshold if their linkage disequilibrium r^2^ was greater than or equal to 0.7 with a SNP with empirical values within the FDR threshold.

**3.8 - Selection of traits for analysis**

Traits were excluded from analysis if they were detected in less than 95% of samples for each tissue region. For each tissue region and trait type (CpG and mRNA) the 95% threshold was determined using total number of analyzable samples for this pairing of region and trait. A probe is considered detected for a sample if the reported Illumina Detection p-value was less than or equal to 0.01.

**3.9 - Polymorphism(s) in Assay Probes**

Sequence variants within the sequence of the probe used to assay individual traits may cause differential hybridization and inaccurate expression and possible methylation measurements. To exclude this confound, the sequences of probes with significant correlation to a trait were examined for the presence of polymorphisms using CEU HapMap data, and, if present, that QTL was removed from the result set.

**3.10 - Relative number and strength of QTLs for different trait types**

To gauge whether QTLs are more prevalent for DNA Methylation or mRNA traits we undertook a resampling analysis using 100 individuals that were assayed for all trait types and randomly selecting 100 CpG and mRNA traits at each sampling. This resampling analysis was performed 10000 times using these 100 individuals, so the same 1,507,278 SNPs are tested at each sampling and the same number of traits are tested per assay type to equalize the number of traits being tested of the ~27,000 CpGs and ~8,000 mRNAs. The resampling analysis was performed using only the frontal cortex samples. The random selection of probes from each assay type was based on probes that were detected in 95% of the frontal cortex samples and were probes that do not contain polymorphisms within the probe sequences, previous section. For this resampling analysis an empirical p-values was not computed and instead a p-value threshold was set at 1-e08 which is less than the Bonferroni adjusted p-value for the number of SNPs being tested per resampled trait. The results of this analysis show on average that 2.34% of CpG sites and 1.99% of mRNAs significantly associate with QTLs. Where each mRNA correlated with twice as many SNPs, on average, than the CpG sites did. However the average strength of the QTLs correlated with mRNAs was equal to CpGs. Frequency Histograms and means from resampling results are show in Figure S6.

**3.11 - Replicated eQTLs**

To identify eQTLs that have previously been reported, we considered the results from studies within the Pritchard Lab EQTL Browser (http://eqtl.uchicago.edu/). Of our 282 mRNA transcripts with a cis eQTL detected in at least one tissue, 149 of these have also been seen in at least one or more of the other studies, Table S2 contains information about these mRNA transcripts. For the 53 mRNA transcripts with a cis eQTL that we detect in all four of brain regions, 37 of these have been in at least one or more of the other studies. Overlap with findings in lymphoblast cell line HapMap samples (Stranger 2007) is 76 transcripts, in cortical samples (Myers 2007) the overlap is 19 transcripts and in liver (Schadt 2008) the overlap is 108 transcripts. While it may be discouraging that the overlap with findings from the Myers cortical samples is not larger, we believe this is a function of the coverage of genetic variance included in the analysis. The current study includes genotypes from 550K (~1.65 million after imputation and selection) SNPs where as the Myers study included 366,140 SNPs; the Schadt study used 782,476 SNPs and the Stranger study; used ~2.2 million SNPs. So ~53% of our cis eQTLs have also been seen in at least one other study; this does not take under consideration differences in the tissues, assay platforms, analysis methods and annotations used in these studies.

**References**

Li, Y., and Abecasis, G.R. (2006). Mach 1.0: rapid haplotype reconstruction and missing genotype inference. Am. J. Hum. Genet. *S79*, 2290.

The International HapMap Consortium. A Haplotype Map of the Human Genome.

Nature 437, 1299-1320. 2005.

Stranger BE, Nica AC, Forrest MS, Dimas A, Bird CP, Beazley C, Ingle CE, Dunning M, Flicek P, Koller D, Montgomery S, Tavaré S, Deloukas P, Dermitzakis ET. Population genomics of human gene expression. Nat Genet. 2007 Oct;39(10):1217-24.

Myers, A.J., Gibbs, J.R., Webster, J.A., Rohrer, K., Zhao, A., Marlowe, L., Kaleem, M., Leung, D., Bryden, L., Nath, P., Zismann, V.L., Joshipura, K., Huentelman, M.J., Hu-Lince, D., Coon, K.D., Craig, D.W., Pearson, J.V., Holmans, P., Heward, C.B., Reiman, E.M., Stephan, D., and Hardy, J. (2007) A survey of genetic human cortical gene expression. Nat Genet. *39*, 1494-1499.

Schadt EE, Molony C, Chudin E, Hao K, Yang X, Lum PY, Kasarskis A, Zhang B, Wang S, Suver C, Zhu J, Millstein J, Sieberts S, Lamb J, GuhaThakurta D, Derry J, Storey JD, Avila-Campillo I, Kruger MJ, Johnson JM, Rohl CA, van Nas A, Mehrabian M, Drake TA, Lusis AJ, Smith RC, Guengerich FP, Strom SC, Schuetz E, Rushmore TH, Ulrich R. Mapping the genetic architecture of gene expression in human liver. PLoS Biol. 2008 May 6;6(5):e107.

Purcell, S., Neale, B., Todd-Brown, K., Thomas, L., Ferreira, M.A., Bender, D., Maller, J, Sklar, P., de Bakker, P.I., Daly, M.J., and Sham, P.C. (2007) PLINK: A Tool Set for Whole-Genome Association and Population-Based Linkage Analyses. Am. J. Hum. Genet. *81*, 559-575.

van der Brug, M.P., Blackinton, J., Chandran, J., Hao, L.Y., Lal, A., Mazan-Mamczarz, K., Martindale, J., Xie, C., Ahmad, R., Thomas, K.J., Beilina, A., Gibbs, J.R., Ding, J., Myers, A.J., Zhan, M., Cai, H., Bonini, N.M., Gorospe, M., and Cookson, M.R. (2008) RNA binding activity of the recessive parkinsonism protein DJ-1 supports involvement in multiple cellular pathways. Proc. Natl. Acad. Sci. USA *105*, 10244-10249.

Saeed AI, Sharov V, White J, Li J, Liang W, Bhagabati N, Braisted J, Klapa M, Currier T, Thiagarajan M, Sturn A, Snuffin M, Rezantsev A, Popov D, Ryltsov A, Kostukovich E, Borisovsky I, Liu Z, Vinsavich A, Trush V, Quackenbush J. TM4: a free, open-source system for microarray data management and analysis. Biotechniques. 2003 Feb;34(2):374-8.

Eisen, M.B., P.T. Spellman, P.O. Brown, and D. Botstein (1998) Cluster analysis and display of genome-wide expression patterns. Proceedings of the National Academy of Sciences USA 95:14863-14868.

Churchill, G A, and R W Doerge. “Empirical threshold values for quantitative trait mapping.” Genetics 138, no. 3 (November 1994): 963-71.

Bibikova, Marina, Zhenwu Lin, Lixin Zhou, Eugene Chudin, Eliza Wickham Garcia, Bonnie Wu, Dennis Doucet, et al. “High-throughput DNA methylation profiling using universal bead arrays.” Genome Research 16, no. 3 (March 2006): 383-93.

Carrel, Laura, and Huntington F Willard. “X-inactivation profile reveals extensive variability in X-linked gene expression in females.” Nature 434, no. 7031 (March 17, 2005): 400-4.

Workman C., Jensen L.J., Jarmer H., Berka R., Gautier L., Nielser H.B., Saxild H.H., Nielsen C., Brunak S., Knudsen S. (2002). A new non-linear normalization method for reducing variability in DNA microarray experiments. Genome Biol, 3(9):research0048.

Schadt, E.E., Li, C., Ellis, B., Wong, W.H. (2001). Feature extraction and normalization algorithms for high-density oligonucleotide gene expression array data. J. Cell. Biochem. Suppl. (Suppl. 37) 120–125.

Tseng, G.C., Oh, M.K., Rohlin, L., Liao, J.C., Wong, W.H. (2001). Issues in cDNA microarray analysis: quality filtering, channel normalization, models of variations and assessment of gene effects. Nucleic Acids Res. 29, 2549–2557.
